# Metagenomic insights into inhibition of soil microbial carbon metabolism by phosphorus limitation during vegetation succession

**DOI:** 10.1093/ismeco/ycae128

**Published:** 2024-10-23

**Authors:** Haocai Wang, Hang Wang, Thomas W Crowther, Kazuo Isobe, Peter B Reich, Ryunosuke Tateno, Weiyu Shi

**Affiliations:** Chongqing Jinfo Mountain Karst Ecosystem National Observation and Research Station, School of Geographical Sciences, Southwest University, Chongqing 400715, China; Dianchi Lake Ecosystem Observation and Research Station of Yunnan Province, Kunming 650228, China; Department of Environment Systems Science, Institute of Integrative Biology, ETH Zürich, Zürich 8092, Switzerland; Institute of Ecology, College of Urban and Environmental Sciences, Peking University, Beijing 100871, China; Institute for Global Change Biology and School for Environment and Sustainability, University of Michigan, Ann Arbor, MI 48109, United States; Department of Forest Resources, University of Minnesota, Saint Paul, MN 55108, United States; Hawkesbury Institute for the Environment, Western Sydney University, Penrith, NSW 2751, Australia; Filed Science Education and Research Center, Kyoto University, Kyoto 606-8502, Japan; Chongqing Jinfo Mountain Karst Ecosystem National Observation and Research Station, School of Geographical Sciences, Southwest University, Chongqing 400715, China

**Keywords:** vegetation succession, soil metagenomics, microbial functional genes, phosphorus limitation, microbial carbon metabolism

## Abstract

There is growing awareness of the need for regenerative practices in the fight against biodiversity loss and climate change. Yet, we lack a mechanistic understanding of how microbial community composition and functioning are likely to change alongside transition from high-density tillage to large-scale vegetation restoration. Here, we investigated the functional dynamics of microbial communities following a complete vegetation successional chronosequence in a subtropical zone, Southwestern China, using shotgun metagenomics approaches. The contents of total soil phosphorus (P), available P, litter P, and microbial biomass P decreased significantly during vegetation succession, indicating that P is the most critical limiting nutrient. The abundance of genes related to P-uptake and transport, inorganic P-solubilization, organic P-mineralization, and P-starvation response regulation significantly increased with successional time, indicating an increased microbial “mining” for P under P limitation. Multi-analysis demonstrated microbial P limitation strongly inhibits carbon (C) catabolism potential, resulting in a significant decrease in carbohydrate-active enzyme family gene abundances. Nevertheless, over successional time, microorganisms increased investment in genes involved in degradation-resistant compounds (lignin and its aromatic compounds) to acquire P resources in the litter. Our study provides functional gene-level insights into how P limitation during vegetation succession in subtropical regions inhibits soil microbial C metabolic processes, thereby advancing our understanding of belowground C cycling and microbial metabolic feedback during forest restoration.

## Introduction

Vegetation restoration is pivotal in mitigating climate change and enhancing the conservation and protection of biodiversity and ecosystem services [1, 2]. Natural regeneration, which allows ecosystems to progress through secondary succession, is receiving increasing attention as an effective natural measurement for restoring degraded habitats [3]. However, soil microbial roles in driving—and responding to—ecosystem succession remain elusive. Integrating the functional response of microbial communities into ecological succession studies can provide insights into the mechanisms that drive succession dynamics and microbial-driven nutrient cycling [4], especially given that the functional potential of microbial metabolism is unclear in the context of nutrient limitation that often occurs in restored ecosystems during long-term succession.

Soil microbial metabolic limitations are partly attributed to nutrient stoichiometric imbalances [5, 6]. When soil carbon (C): nitrogen (N): phosphorus (P) ratios deviate from microbial metabolic demands, microorganisms must adjust their corresponding metabolic functions to acquire limited resources for maintaining elemental stoichiometric balance rather than growth, thereby regulating the rate of C and nutrient cycling [5, 7]. Relative to primary production [8], microbial nutrient limitations thus potentially regulate detrital organic C retention and ultimately the size of terrestrial C pool [9–12]. Given the challenges in ascertaining microbial metabolic limitations in-situ, enzyme activity models have relied heavily on extracellular enzyme stoichiometry theory to assess microbial metabolic characteristics [7, 9, 13–15]. The ecoenzyme vector model is one of the most widely used approaches to interpret ecoenzymatic stoichiometry [16]. However, determining microbial nutrient limitations using ecoenzyme-based stoichiometric approaches remains highly uncertain [17, 18], partly because several commonly assayed enzymes (e.g. β-1,4-glucosidase, β-D-cellobiosidase, leucine aminopeptidase, β-1,4-N-acetylglucosaminidase, and acid/alkaline phosphatase) only represent the potential activities of specific metabolic pathways, overlooking potential gene regulation of specific metabolic pathways crucial to the C, N, and P cycles. Additionally, the lack of accurate ecological enzyme stoichiometric thresholds corresponding to specific nutrient limitations further complicates this determination [19]. In comparison, metagenomic sequencing can provide direct information on the functional gene diversity and abundance of microbial communities, which provides additional evidence for a more precise assessment of the metabolic potential of microorganisms [20].

Although vegetation succession can increase the accumulation of C and N contents in the soil through photosynthesis and N fixation [21–23], P is a relatively stable and scarce element in the soil with its sources mainly back to rock mineralization [24]. There is mounting evidence that P limitation could be as impactful as N limitation in terrestrial ecosystems [8, 25, 26]. The theories of natural succession posit that long-term ecosystem development can concentrate a great proportion of the available P into the slow-turnover pools such as wood and soil organic matter [27], resulting in a decreasing proportion of P being actively recycled within the ecosystem. Thus, natural ecosystems gradually tend to be limited by P availability with vegetation restoration succession, especially in many tropical and subtropical forest areas [8, 28–30]. Microbes play an integral role in soil P cycling, as they mediate bioavailable soil P [31, 32]. Critical microbial P cycling-related genes encode a series of enzymes, synthesizing specific proteins that facilitate soil P transformation [33]. Interestingly, there is evidence that microbial metabolism during vegetation succession is affected by P limitation based on enzyme stoichiometry [34–36], although it remains unclear how key gene families involved in P cycling processes respond to nutrient limitation and how this affects other metabolic activities of microorganisms. In particular, few studies have focused on the role of soil microbial functional genes involved in P cycling processes in controlling soil C dynamics in the context of P limitation during vegetation succession. A recent study suggests that P captured by soil microorganisms constrains ecosystem P recycling and availability for plant uptake, thus limiting the ability of trees to take up additional C [12]. For soil C pools, a key question during long-term vegetation succession is how microbial C metabolic processes adjust in face to soil P limitation due to the increased plant P demand induced by vegetation restoration. Soil microorganisms often acquire C resources by discharging extracellular enzymes to decompose complicated small organic molecules, containing labile or recalcitrant C [37]. The carbohydrate-active enzyme (CAZyme) has recently been employed to explore microbial functional responses to C turnover, and a few studies have identified specific glycoside hydrolases (GH) and auxiliary activity enzymes (AA) that drive C dynamics, associated with the decomposition of polysaccharides and lignin, respectively [38, 39]. However, trends in the microbial CAZyme family induced by nutrient limitation during vegetation succession and its effect on C turnover remain to be elucidated.

In China, the restoration or reforestation of forests has significantly influenced the global greening trend [40]. Linking functional responses to microbial metabolic limitation to vegetation restoration may enable optimal management of restored vegetation and improve the accuracy of model prediction for soil C sequestration potentials in restored subtropical forest ecosystems. Subtropical ecosystems tend to have highly weathered soils with high plant P demand, and thus P is commonly regarded as the most limiting nutrient [8, 25]. Therefore, we hypothesized that during vegetation succession, with an increase in C supply, microbial C metabolism—proxied by “C metabolism functional genes”—is still inhibited due to P limitation resulting from insufficient P resources for elemental balance. In this study, we employed shotgun metagenomics sequencing to analyze the functional attributes of microbial metabolic limitation in five vegetation communities in a typical vegetation restoration area in southwestern China, which are distributed along a restoration gradient with an average stand age ranging from 1 year to >50 years. We explored: (i) what are the changing patterns of functional shifts in microbial communities that occur along the succession time-series, and do shifts in microbial genes affect nutrient cycling and drive succession as feedback? (ii) How does microbial metabolic limitation due to nutrient limitation respond to vegetation succession at the level of functional genes?

## Materials and Methods

### Experimental design and sampling

**Figure 1:**
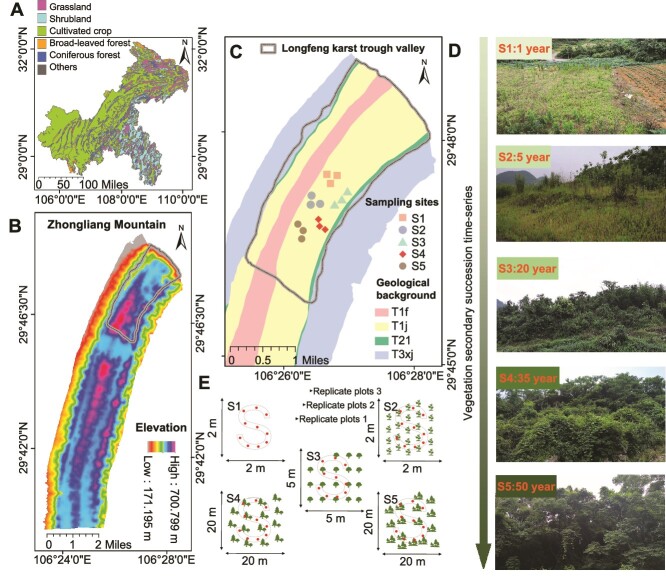
Geographic location and sampling schematic for the study sites in typical vegetation restoration areas in Southwest China. The geographic map displays the study area, Zhongliang Mountain, along with the distribution of vegetation types in Chongqing municipality where it is situated (A). The elevation map of Zhongliang Mountain and the sampling area, Longfeng karst trough, are depicted (B). A geological background map of the study area shows the distribution of our sampling sites (C). The geological formations identified include T1f: Feixianguan formation of the lower Triassic; T1j: Jialingjiang formation of the lower Triassic; T2l: Leikoupo formation of the middle Triassic; and T3xj: Xujiahe formation of the upper Triassic. The sampling sites in this study are all situated in the T1j geologic background area. The landscapes of typical vegetation types, restored on lands of varying abandonment ages and representing the five different stages of vegetation restoration, are selected as our sampling sites in this study (D). Sample plot size and sampling schematic for different stages of vegetation restoration are shown (E).

The study was carried out in the Longfeng karst trough valleys (106°25′ ∼ 106°29′E, 29°45′ ∼ 29°50′N; altitude 171–700 m; humid subtropical monsoon climate) of the northern part of the Zhongliang Mountain located in the northwest of Chongqing, Southwest China (Fig. 1A and B). Since the 1950s, rapid population growth has led to increased food demand, causing widespread destruction of native vegetation [subtropical broad-leaved evergreen forests with *Cinnamomum camphora* (L.) Presl., *Sapium sebiferum* (L.) Roxb., *Celtis sinensis* Pers., *Cyclobalanopsis glauca* (Thunb.) Oerst. as the main tree species) for agriculture. However, unsustainable farming practices have exacerbated rocky desertification, leading to farmland abandonment. Thus, naturally occurring successional stages for these farmlands abandoned at different times have been observed in the region [41].

Sampling was conducted in September 2021, at the peak period for above-ground biomass. The geological backgrounds in the study area all belong to the limestone of the Triassic Jialingjiang Formation (Fig. 1C). Maize was the main crop grown in the region before the farmland was abandoned. The recovery time at different stages of succession was estimated by consulting with local elders and considering land contracts between farmers and the government. Across the successional gradient (Fig. 1D), we established five vegetation restoration stages for the study: (i) abandoned land (abandoned for 1 year, S1); (ii) grassland stage (~5 years after abandonment, S2); (iii) shrubland stage (~20 years, S3); (iv) shrub and arboreal mixed stage (~35 years, S4); and (v) arboreal stage (~50 years, S5). The five successional stages selected represent a typical time course of vegetation recovery in the region, and the site with the older successional time has experienced a pre-successional sequence of vegetation recovery. The sites selected for this study, representing various stages of succession, have all evolved from abandoned farmland through gradual succession. In general, the specific sites for the five successional stages were selected to ensure homogeneous geological backgrounds, similar human cultivation histories, and adherence to the sequence of vegetation restoration. Details of information for each site are listed in Supplementary information Table S1. Three independent replicate plots (with a >50 m buffer zone between each one) were randomly established for each stage, where the distance between any two stages was not <100 m apart and not >1 km apart. Ten soil cores (5.0 cm inner diameter) were randomly collected at a depth of 0–20 cm of each plot in an “S” shape (Fig. 1E), after excluding the litter and humus layers in each plot (except for abandoned land), and they were mixed thoroughly to yield one composite sample. Thus, a total of 15 composite samples were collected (5 succession stages × 3 replicate plots × 1 composite sample mixed from 10 samples of each plot). The soil samples were stored in a portable cooler at 4°C and transported to the laboratory. They were then divided into two subsamples: one was maintained at 4°C for physicochemical analysis, while the other was frozen at −80°C for deoxyribonucleic acid (DNA) extraction. For sampling the litter layer, we initially established five 0.5 m × 0.5 m small plots on each replicate plot. Subsequently, litter from various plants under the forest canopy was manually collected, and the five litter samples from the same plot were combined into one sample.

### Measurement of soil-litter physicochemical properties

Standard testing methods were adopted for the measurement of soil pH, soil water content (SWC), soil bulk density (BD), percentage of clay, silt and sand, total C (TC), soil organic C (SOC), soil dissolved organic C (DOC), soil available P (AP), microbial biomass P (MBP), soil NH_4_^+^ and NO_3_^−^ N, soil total N (TN), soil total P (TP), soil total potassium (TK), litter total potassium (LTK), litter total P (LTP), litter total N (LTN), litter total C (LTC), and the content of hemicellulose, cellulose and lignin in litter, as previously described [42–44]. LC/P, LN/P, and LC/N are denoted as abbreviations for LTC/LTP, LTN/LTP, and LTC/LTN ratio, respectively. More details of the measurement of soil and litter physicochemical properties are provided in the Supplementary methods section of the Supplementary information.

### Deoxyribonucleic acid extraction, deoxyribonucleic acid sequencing, metagenomic processing, and functional annotation

Soil DNA was extracted in triplicate from 0.5 g of fresh soil sample using the FastDNA spin kit for soil (MP Biomedicals, Cleveland, USA), following the manufacturer’s instructions. The quality and integrity of the DNA extracts were assessed using a NanoDrop 2000 spectrophotometer (Thermo Scientific, USA). The extracted microbial DNA was processed to construct metagenomic shotgun sequencing libraries with insert sizes of ~400 bp using the Illumina TruSeq Nano DNA LT Library Preparation Kit. Each library was sequenced by HiSeq 4000 PE150 platform at LC-Bio Technology Co., Ltd (Hangzhou, China). The raw sequence data generated in this study have been deposited on the National Center for Biotechnology Information (NCBI), with the accession number PRJNA1080685. For further details on sequencing and bioinformatic analysis, see the Supplementary methods section of the Supplementary information (Figure. S1, Table S2).

### Statistical analyses

#### The succession of soil and litter profiles and functional attributes of microbial carbon–nitrogen–phosphorus cycling

Principal component analysis was first conducted using the “FactoMineR” package (https://github.com/husson/FactoMineR) to analyze variations in litter and soil properties across vegetation succession stages. Non-metric multidimensional scaling (NMDS) analysis, based on Bray–Curtis dissimilarity, assessed the differences in microbial functional attributes, with significance tested performed by analysis of similarity (ANOSIM). Additionally, differences in functional gene abundance across successional stages were evaluated using one-way analysis of variance (ANOVA; *P* < .05, LSD test). Ordinary least squares (OLS) regression analysis explored the relationship between succession time and the abundance of selected functional genes. Finally, a heatmap depicted the abundance of selected functional genes across all succession stages.

#### Co-occurrence network among microbial carbon, nitrogen, and phosphorus cycling functional genes, as well as the annotated taxa

To assess interactions between microbial functional genes involved in C degradation, N, and P cycling across all successional stages, the “ggClusterNet” package (https://github.com/taowenmicro/ggClusterNet) was utilized to generate an interaction network. Robust correlations (Spearman’s *ρ* > 0.7, FDR-adjusted *P* < .05) were identified for all pairwise combinations of these genes [45]. Each node in the network represents a functional gene, with edges indicating strong and significant correlations between genes. Additionally, negative edge correlation networks were constructed separately to highlight relationships between C degradation and P cycling genes, as well as between C degradation and N cycling genes. This facilitated the examination of genetic-level relationships in response to nutrient stoichiometric imbalances during vegetation succession. Similarly, we employed the same criteria to construct a metacommunity co-occurrence network encompassing all taxonomic groups (species level) annotated for C degradation, N, and P cycling functional genes. Two sub-networks focused solely on significant negative correlations between C degradation-N cycle and C degradation-P cycle taxa. Rare taxonomic groups (relative abundances <0.01%) were excluded to streamline the dataset. Co-occurrence patterns were analyzed using the “igraph” package’s induced subgraph function and visualized using the interactive Gephi platform (https://gephi.org/).

### Drivers of soil microbial changes in carbon, nitrogen, and phosphorus functional attributes

Initially, we conducted variance partitioning analysis (VPA) using the “vegan” package [46] to determine the relative importance of functional gene annotation taxa, vegetation, and soil properties on changes in C degradation, N, and P cycling functional genes with vegetation succession. Before performing VPA, we calculated *α* and *β* diversity indices for the annotated taxa involving C degradation, N, and P cycling functional genes and then selected richness and Shannon indices, as well as the first dimension of NMDS and MBP, as indicators of microbial composition. We further employed the random forest algorithm from the “RandomForest” package [47] to determine which variables predominantly affect microbial C degradation, N and P cycling genes, and the significances of each predictor and model were assessed with 1500 permutations using the “rfPermute” (https://github.com/EricArcher/rfPermute) and “A3” (https://rdrr.io/cran/A3/) packages, respectively. Furthermore, a partial least squares-path model (PLS-PM) was constructed to further elucidate the contribution of key variables to functional genes for C degradation, N and P cycling using the “plspm” package (https://github.com/gastonstat/plspm). To ensure the explanatory power of the model and the reliability of the results, we only retain key variables with loading values >0.7 during the execution of PLS-PM and categorize them into different attribute groups.

All above-mentioned statistical analyses were done using R environment (v4.2.1, https://www.r-project.org/).

## Results

### Patterns of soil microbial communities, functional attributes, and C degradation potential during vegetation succession

Throughout successional time, the dominant vegetation, litter properties, and soil physicochemical characteristics showed progressive shifts (Table S1**,**Figs S2 and S3). Bacteria comprised >90% of total soil microbial abundance (Fig. S4A) and showed significant variations across five successional stages (ANOVA, *P* < .05; Table S7) compared to fungal and archaeal communities. Soil microbial α-diversity, both taxonomic and functional, increased with succession time, while *β*-diversity varied across successional stages (ANOSIM, *P* = .001; Fig. S4B–E).

**Figure 2:**
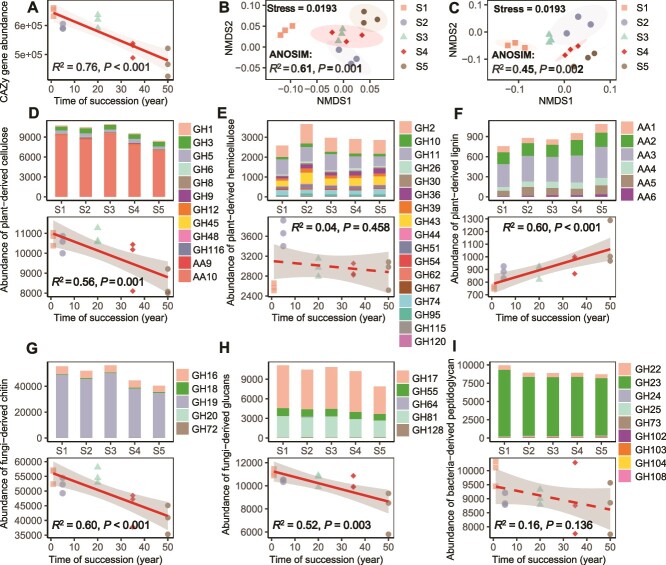
Changes in the composition and abundance of genes from the CAZyme database involved in C decomposition with vegetation succession time. Regression analysis of the association between years of succession and the abundance (indicated by TPM, transcripts per kilobase per million mapped reads) of all CAZy genes (A). NMDS based on the Bray–Curtis distance of pairwise samples according to the abundance of genes involved in the plant-derived C decomposition (B) and microbial-derived C decomposition (C) at all successional stages. ANOSIM was used to examine differences in C-degrading genes involved with plant- and microbial-derived C decomposition across different stages of succession. Regression analysis of the association between years of succession and the abundance of genes responsible for plant-derived cellulose decomposition (D), genes responsible for plant-derived hemicellulose decomposition (E), genes responsible for plant-derived lignin decomposition (F), genes responsible for fungi-derived chitin decomposition (G), genes responsible for fungi-derived glucans decomposition (H), and genes responsible for bacteria-derived peptidoglycan decomposition (I). The solid lines indicate the fitted ordinary least-squares model and the gray areas represent the 95% confidence intervals.

**Figure 3:**
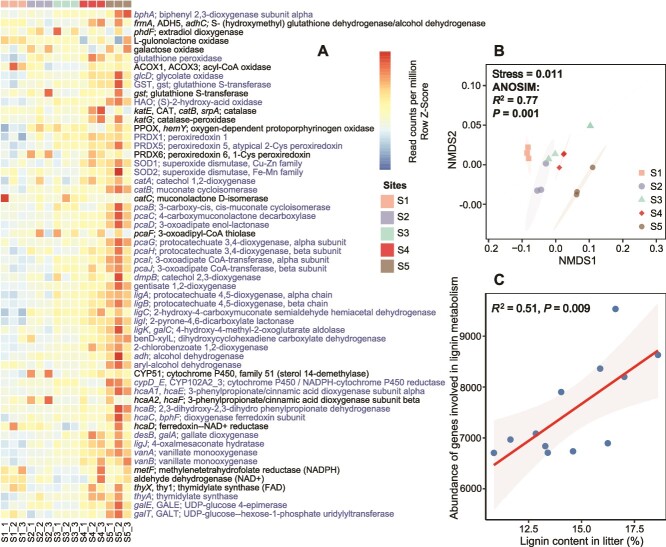
Differences in composition and abundance of catabolic genes involved in lignin depolymerization, oxidative stress response, and catabolism of lignin-derived aromatic compounds in the KEGG database with time of vegetation succession. Heatmaps of normalized abundance values (row Z-scored) were obtained using the number of sequences annotated for the 58 enzyme-encoding genes involved in the transformation of lignin and its derived aromatic compounds in each microbial consortium (A). Genes with significant regression relationships with succession time (*P* < .05) are highlighted. Microbial functional differences involved in the ligninolytic genes at different successional stages were determined by NMDS based on Bray–Curtis distance (B). An ANOSIM was used to examine differences in the functional profiles of all genes involved in lignin decomposition for all successional stages. The linear regressions between abundance of genes involved in lignin decomposition and lignin content in litter (C). Please see Table S4 for the detailed explanation of these genes.

The relative abundance of CAZy genes decreased significantly with successional time (*R*^2^ = 0.76, *P* < .001; Fig. 2A). Among CAZymes (Table S3), genes encoding glycoside hydrolases (GH) and auxiliary enzymes (AA) varied significantly across successional stages (Fig. 2B and C; Table S8). Gene abundances related to cellulose, chitin, glucans, and peptidoglycan decomposition decreased significantly over succession time (Fig. 2D, G, and H). Although the decrease in genes degrading hemicellulose and glucans was not significant, it also showed a downward trend (Fig. 2E and I). In contrast, genes encoding plant lignin-converting enzymes increased significantly (*R*^2^ = 0.60, *P* < .01; Fig. 2F). NMDS analysis of 58 catabolic genes related to lignin degradation, oxidative stress, and lignin-derived aromatic compound metabolism revealed that they differed across successional stages (Fig. 3B) and that the abundance of 40 genes increased significantly over time (Fig. 3A; Fig. S5). Lignin degradation gene abundance was positively correlated with litter lignin content (*R*^2^ = 0.51, *P* = .009; Fig. 3C). Microbial taxa (phylum level) involved in plant- and microbial-derived component degradation varied across successional stages (Fig. S6). Taxonomic annotation of the 58 KEGG genes showed patterns similar to CAZyme composition and succession dynamics (Fig. S7).

### Restoration of microbial nitrogen cycling potential during vegetation succession

**Figure 4:**
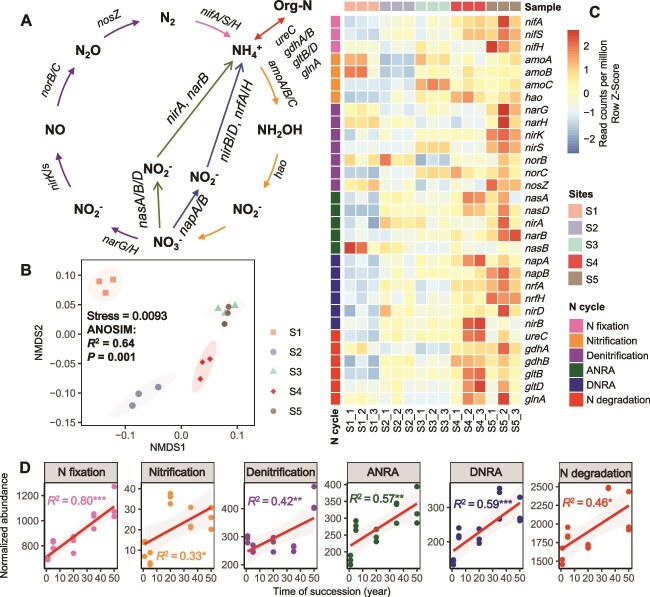
Differences in the abundance (TPM) of N cycle functional genes at different successional stages. Diagram depicting the different N cycling processes based on metagenomic sequencing (A). Microbial functional differences involved in the soil N cycle at different successional stages were determined by NMDS based on Bray–Curtis distance (B). An ANOSIM was used to examine differences in the functional gene profiles involved in the N cycle at different successional stages. Heatmaps displaying variations in the normalized abundance values (row Z-scored) of functional genes associated with N cycling in the KEGG database were generated from our metagenomic data across all successional stages (C). OLS regression analysis was conducted to examine the relationships between years of succession and the abundance of selected functional genes involved in the N cycling: nitrogen fixation, nitrification, denitrification, ANRA, DNRA, and N degradation (D). Significant differences (*P* < .05) between the successional stages were indicated by different lowercase letters, determined using a one-way ANOVA followed by an LSD test (^*^P < .05, ^*^^*^P < .01, ^*^^*^^*^P < .001).

Six N cycling pathways were analyzed, including N_2_ fixation, nitrification, denitrification, dissimilatory nitrate nitrogen reduction to ammonium nitrogen (DNRA), assimilatory nitrate nitrogen reduction (ANRA), and organic N metabolism (Fig. 4A, Table S5). The results revealed significant differences in N cycling gene families across different successional stages (Fig. 4B, Table S9). Heatmap and OLS regression analyses showed that such differences were mainly manifested as a trend of significant enrichment of N cycle gene abundance with successional time series (Fig. 4C and D). Additionally, the composition and diversity of functional groups involved in N cycling changed significantly during secondary succession (Figs S8 and S9).

### Changes in microbial phosphorus cycling potential during vegetation succession

**Figure 5:**
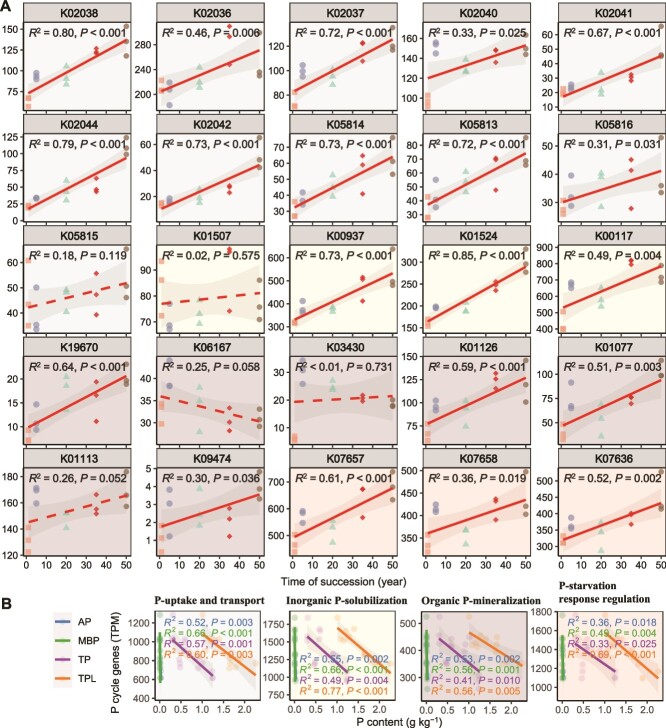
Microbial-driven P-cycling potential as depicted by metagenomic sequencing at different successional stages. OLS regression analysis was conducted to explore the relationships between years of succession and the abundance of selected functional genes of microorganisms involved in soil P cycling: genes coding for P-uptake and transport, genes coding for inorganic P-solubilization, genes coding for organic P-mineralization, genes coding for P-starvation response regulation (A). OLS regression analysis between soil P cycling functional gene abundance (TPM) and total soil P (TP), soil available P (AP), litter P contents (LTP), and microbial biomass P, respectively (B). Please see Table S6 for a detailed explanation of these genes.

To investigate microbial P-cycling gene dynamics during vegetation succession, we analyzed 25 P-related functional genes, including those for P-uptake, transport, inorganic P-solubilization, organic P-mineralization, and P-starvation response (Table S6). Among these, 21 genes showed significant variations across successional stages (ANOVA, *P* < .05, LSD test; Table S10). OLS regression revealed that the majority of P cycling gene abundances were significantly positively correlated with successional time (Fig. 5A) and negatively correlated with AP, MBP, TP, and LTP content (Fig. 5B). Microbial *α*-diversity in the four P cycling pathways increased significantly over succession (Fig. S10A and B), with significant differences in community structure observed across successional stages (ANOSIM, *R*^2^ = 0.99, *P* = .001; Fig. S10C). Proteobacteria consistently dominated the taxa involved in P cycling across all stages (Fig. S11).

### Microbial functional networks associated with carbon degradation, nitrogen-cycling, and phosphorus-cycling

**Figure 6:**
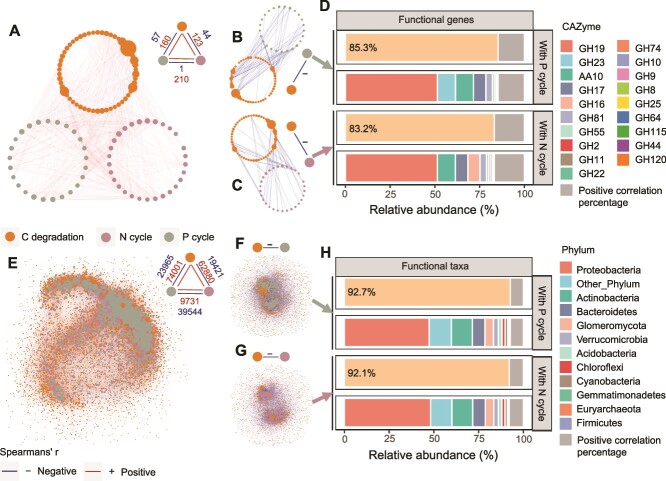
Occurrence networks of genes involved in C degradation, N and P cycling, as well as associated species in soils. Overview of networks distributed by C-degradation genes, N-cycling genes, P-cycling genes (A), and their co-occurrence patterns (E) in corresponding taxonomically annotated communities for all successional stage metagenomic. A connection indicates a strong (Spearman’s *P* > .7) and significant (FDR-corrected *P* < .05) correlation. The size of each node is proportional to the degree between two individual nodes. The composition and relative proportions of C-degradation genes exhibiting significant negative correlations with P-cycling genes and N-cycling genes in the network relationships were examined (B–D). Overview of co-occurrence patterns of taxonomically annotated communities encoding C-degradation genes, N-cycling genes, and P-cycling genes in all successional stage metagenomic (E). The composition and proportion of annotated taxa associated with C-degradation genes that exhibited a significant negative correlation with annotated taxa associated with P-cycling genes and those associated with N-cycling genes were examined respectively (F–H).

Based on the co-occurrence networks among functional genes, most of the C degradation genes exhibited a positive correlation with N and P cycling functional genes (Fig. 6A). Subsequently, we generated subnetworks for microbial C degradation genes, retaining only negative correlations with N and P cycling functional genes (Fig. 6B and C). We then separately calculated the composition and abundance proportion of C degradation genes negatively correlated with N and P cycling functional genes among all C degradation genes (Fig. 6D). A total of 19 C degradation genes, including GH19 (chitinase), GH23 (lysozyme/peptidoglycan lytic transglycosylase), and AA10 (lytic polysaccharide monooxygenase), were negatively correlated with N and P cycling functional genes. Despite constituting a minor proportion (35%) of all genes (19/54), their relative abundances within the total abundance of all C degradation genes were remarkably high at 83.2% and 85.3%, respectively (Fig. 6D).

We constructed a co-occurrence network among microbial taxa involved in C degradation, N, and P cycling functions (Fig. 6E). Subnetwork analyses showed negative correlations between C degradation-related microorganisms and those associated with N and P cycling (Fig. 6F and G), indicating a potential inhibition of microbial C degradation by N and/or P cycling functions during vegetation succession, with inhibition ratios as high as 92% and 93% relative abundance, respectively (Fig. 6H).

### Drivers of soil microbial metabolic function changes during vegetation succession

**Figure 7:**
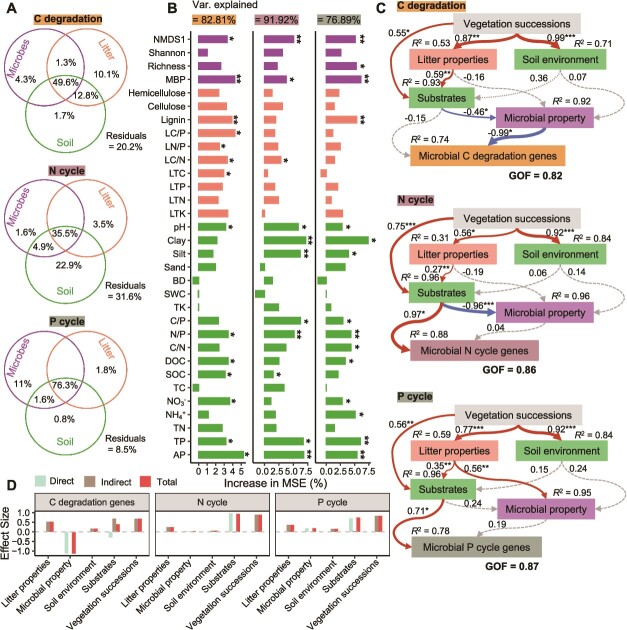
Driving forces of altered soil microbial C, N, and P functional genes during vegetation succession. Variation partitioning analysis showing the effects of litter, soil, and microbial community attributes on microbial C, N, and P functional genes (A). Random forest modeling identifies the contribution of each predictor variable in explaining changes in the microbial C, N, and P cycling genes during vegetation succession (B). MSE, mean squared error. ^*^ represents significant effects at *P* < .05, ^*^^*^ represents significant effects at *P* < .01. Partial least squares path modeling (PLS-PM) untangles primary pathways linking key drivers to the functional potential of soil microbial C, N, and P cycling in vegetation succession, as well as their impacts on soil microbial C, N, and P cycling potential (C, D). The widths of the arrows indicate the strength of the casual relationship. Numbers on the arrow indicate the standardized path coefficients. *R*^2^ indicates the variance of the dependent variable explained by the models. GOF: the goodness of fit of the model. ^*^ represents significant effects at *P* < .05; ^*^^*^ represents significant effects at *P* < .01; ^*^^*^^*^ represents significant effects at *P* < .001.

To identify factors driving changes in soil microbial metabolic functions during vegetation succession, we conducted variance partitioning and random forest analyses, focusing on functional microbial taxa, soil physicochemical characteristics, and litter chemical properties. These factors collectively explained 49.6% of C degradation, 35.5% of N cycling, and 76.3% of P cycling gene abundance variations (Fig. 7A). Random forest analysis further showed that these variables explained 82.8% of the variation in microbial C metabolism, 91.9% in N cycling, and 76.9% in P cycling (Fig. 7B). We found that nutrient indicators related to soil P content (AP, TP, MBP), organic C content (SOC, DOC, LTC), and C:N:P ratios (LC/P, LN/P, N/P) were key drivers of changes in microbial C degradation functions during vegetation succession. Similarly, multiple nutrient factors also drove shifts in microbial genes involved in N and P cycling.

Finally, based on the results of the random forest model, we revealed potential pathways driving changes in soil microbial metabolic functions during vegetation succession by constructing a PLS-PM model (Fig. 7C and D). The model fit indices (goodness of fit, GOF) for C degradation, N cycling, and P cycling were 0.82, 0.86, and 0.87, respectively, indicating the reliability of the model outcomes. The results further demonstrated that soil AP and TP strongly inhibited microbial C degradation potential by exerting a significant negative direct impact on microbial MBP and community structure (NMDS1; Fig. 7C and D). Additionally, since NO_3_^−^ had a loading value <0.7 in the PLS-PM model for microbial C degradation genes (Fig. S12B) and showed no significant correlation with C degradation gene abundance (Fig. S12A), we confirmed that P limitation, rather than N, was the key factor driving the significant decline in microbial C degradation potential during vegetation succession. In contrast to the processes driving microbial C degradation potential, the abundance of N- and P-cycling genes during vegetation succession is directly and significantly positively influenced by the substrate properties (Fig. 7C and D).

## Discussion

### Patterns of microbial functional genes and nutrient limitation during vegetation succession

In line with previous studies [48–50], vegetation restoration during vegetation succession enhanced organic matter input and substrate effectiveness, notably increasing SOC, DOC, and NH_4_^+^ concentrations (Fig. S3). Furthermore, we found that vegetation succession processes facilitated soil texture improvement (Fig. S3). In subtropical regions, enhanced activities of plant roots and soil microorganisms during the long-term natural vegetation succession and restoration process may contribute to soil texture improvement, resulting in a decrease in clay content and an increase in sand and silt content in the surface soil layer. Nonetheless, P concentrations in soil, litter, and microbial biomass declined significantly with succession, leading to significant increases in C/P and N/P ratios (Fig. S3). According to Sprengel–Liebig’s law of the minimum [51], P is the primary limiting nutrient for microbial metabolism. Based on the N/P ratio of vegetation directly reflecting nutrient limitation (N/P limitation) at the community level (i.e. the N/P threshold hypothesis) [52, 53], N might have briefly constituted the main limiting nutrient for plant growth early in succession. However, in prolonged and complex succession, microbial N cycling function significantly recovers, potentially benefiting from biological N fixation (Fig. 4). A previous study indicated that N fixation plays an important role in maintaining soil N richness in late-successional forests [54]. Consequently, N ceases to be the limiting nutrient shortly after the commencement of vegetation succession (Figs 7 and S12). Moreover, when ecosystems shift from relative N limitation to P limitation, plants sometimes reabsorb more P than N before senescing tissue becomes litter to maintain their N/P stoichiometric balance. The relatively low N absorption by plants in P-deficient habitats provides more N to support microbial production of phosphatases [55]. Our results align with studies indicating forest succession enriches soil N but leads to P limitation [54]. Indeed, P limitation significantly affects primary production and ecosystem processes in various terrestrial ecosystems [56], with broader and stronger limitations on plant productivity than previously estimated [29].

The shifts in microbial functional genes reflect the intrinsic drivers of the soil P cycling process. Our results showed that microbial functional genes involved in P cycling were affected by P depletion, and their gene abundance increased significantly with successional time (Fig. 5A), and were significantly negatively correlated with soil TP, AP, litter P content, and microbial biomass P (Fig. 5B). The results of PLS-PM analysis also showed that vegetation succession had a significant negative effect on soil AP and TP (Fig. S12C), altering the soil C:N:P ratio and thereby regulating microbial P cycling functional genes (Fig. 7C). Microbial solubilization of calcium and mineral phosphates attributed to acidification of the periplasmic space [57]. In this study, the gene encoding inorganic P solubilization was one of the most abundant P cycle-related genes in the dataset, and the significant increase in its abundance with successional time may be evidence of an increased microbial potential for mineral P solubilization under P limitation. In addition, when inorganic P is limited, soil organic matter may be the most important source of P for microorganisms [9]. According to the model hypothesized by McGill and Cole [58], soil P mineralization, i.e. decomposition of P-containing organic matter, is driven by P stress and is not inhibited by organic matter mineralization. When microbes are P-limited, they preferentially hydrolyze organic molecules with low C/P ratios to release P [59]. The high abundance of P signaling and P-regulated genes in the dataset and the significant increase in their abundance with successional time reflect a strong signal of microbial P starvation at the genetic level. Overall, our findings demonstrate that P was the primary limiting nutrient during vegetation succession, highlighting the enhanced microbial potential for P cycling during this successional process.

### Phosphorus limitation inhibits microbial carbon metabolism potential during vegetation succession

Vegetation restoration has been reported to produce more C sources to feed soil microorganisms, resulting in lower C limitation for microorganisms [60]. Since soil microbial activity is required to maintain elemental stoichiometric balance and the nutrient supply environment remains homeostatic under different types of vegetation [61], microbial C metabolism may be compromised under increased P limitation, despite a significant increase in organic C content with vegetation succession. The results of this study showed that the relative abundance of CAZy genes decreased significantly with increasing successional time (Fig. 2A), which is consistent with the previous findings of Zhong *et al.* [62], who also observed a decrease in soil microbial C cycling gene abundance during secondary succession, which was linked to reduced litter decomposition ability. Despite previous studies using laboratory substrate-added incubations, field fertilization experiments, or stoichiometry-based models have demonstrated that P limitation can suppress soil C release [10] or the soil priming effect [63], recent observations suggest that in substrate-addition experiments, certain microbial communities relying on enzyme-producing organisms for resources but not producing these enzymes themselves may still experience resource limitations [7]. Therefore, caution is needed when applying enzymatic methods in disturbed systems, such as nutrient-enriched agricultural systems or short-term incubation experiments [7]. Additionally, although some studies have investigated microbial P limitation during forest succession using ecoenzymatic stoichiometry [64, 65], they have not fully explored how P limitation affects other nutrient cycles, such as C and N. This is partly because ecoenzymatic stoichiometry infers microbial metabolic limitations by measuring a few key enzymes associated with C, N, and P acquisition, which only indicate potential activity in specific metabolic pathways and do not directly capture the underlying gene regulation. In comparison, metagenomics sequencing, which identifies specific genes and pathways directly involved in C, N, and P cycling, provides higher resolution and broader functional coverage for assessing microbial nutrient limitations in natural ecosystems, allowing us to clarify the inhibitory effect of P limitation on microbial C decomposition potential at the genetic level (Figs 6 and 7). Specifically, we observed that the relative abundance of C decomposition genes (taxa) that were significantly negatively correlated with P cycling was dominant among all C decomposition genes (taxa), despite their numerical disadvantage compared to those that were significantly positively correlated (Fig. 6). The random forest and PLS-PM models indicate that during secondary succession, soil AP and TP inhibited microbial C degradation potential by regulating functional groups and MBP (Figs 7 and S12). Based on substrate limitation theory [66], microorganisms under increased P limitation during vegetation succession invest more energy to acquire P, while weakening C decomposition potential to reach the coupled elemental requirements of microbial stoichiometric homeostasis [5, 67]. The shift from high abundance of genes encoding enzymes for C-degradation to high abundance of genes encoding enzymes for P-acquisition with the vegetation succession time-series supports resource allocation theory [68], which implies that microorganisms can reduce stoichiometric imbalances in their resources by increasing the secretion of specific enzymes required to mine scarce nutrients, manifested in this study as a shift in the abundance of genes encoding the synthesis of relevant enzymes. In addition, in this study, we observed no significant correlation between soil-effective N (NO_3_^−^) and C degradation gene abundance (Fig. S12A), and the loading value in the PLS-PM model was <0.7 (Fig. S12B), suggesting that N is not a direct factor driving the decline in microbial C degradation potential. Recent studies have confirmed that increased soil N content (N deposition) in subtropical forests inhibits SOC decomposition by enhancing P limitation [63]. Overall, we suggest that synergistic responses to stoichiometric imbalances in functional genes encoding C degradation and P cycling in soil microbial communities result in lower microbial threshold element ratios, which inhibit microbial C metabolism potential.

### Enhanced lignin degrading potentials with successional time

Across the study, soil microorganisms that utilize C sources from plant and microbial biomass have multiple microbial CAZymes that are involved in the degradation of both biomasses (Fig. 2). This finding supports the previous view that soil microbes are major consumers of simple and recalcitrant substrates [69]. However, our results showed that the community composition-based potential for degradation of plant-derived cellulose and hemicellulose, fungal-derived chitin and glucan, and bacterial-derived peptidoglycan showed a decreasing trend with successional time (Fig. 2), while the degradation potential of plant-derived lignin increased significantly with successional time (Figs 2 and 3). This trend, apart from being influenced by microbial P limitation, also mirrors the complexity and molecular diversity of soil organic matter pool during vegetation succession [70]. Specifically, we observed that changes in abundance of plant-derived cellulose, hemicellulose, and lignin degradation genes with successional time were consistent with changes in cellulose, hemicellulose, and lignin content in litter (Figs 2 and S3), which, in combination with a significant increase in abundance of genes involved in lignin depolymerization and the catabolism and metabolism of lignin-derived aromatic compounds in the KEGG database with successional time (Fig. 3), suggesting that the degree of organic matter degradation difficulty of plant-derived components is increasing with successional time.

Previous studies have also observed that the cellulose and hemicellulose content of litter material across the successional stages was highest, while the lignin content was the lowest, at the beginning of succession [71]. In the later stages of succession, forests typically feature a higher prevalence of woody vegetation, whereas in the early stages of succession, grasslands and shrublands may contain a richer proportion of cellulose and hemicellulose. These distinctions in vegetation types can alter the quality of C sources within the soil, consequently influencing the C-degrading functionality of microbial communities. It is suggested that the input of complex plant-derived C (e.g. cellulose) strengthens the microbial metabolic demand for P to a much greater extent than that of labile plant-derived C (e.g. glucose) [63]. Thus, increased litter lignin content during vegetation succession may have further stimulated the microbial metabolic demand for P. However, if soil P limitation inhibits microbial metabolic activity and lignin inputs increase microbial metabolic demand for P, microbes may still be required to increase further their investment in degrading resistant compounds to acquire litter P resources to meet increased P demand under conditions of more complex substrate chemistry (lignin and its aromatic compounds) and depletion of soil inorganic P [72, 73].

Overall, substrate quality has a differential effect on microbial metabolic demand for P, which helps to explain the microbial C metabolic potential in response to P limitation. Given that changes in plant composition during vegetation succession can alter the quality of plant-derived C inputs, we suggest that future studies consider substrate quality when assessing how enhanced P limitation with succession time affects microbial C metabolism potential.

## Conclusion

Our study demonstrated that P is the key limiting nutrient driving the metabolic potential of soil microbial communities during vegetation succession, leading to a significant increase in gene abundances regulating P uptake and transport, inorganic P solubilization, organic P mineralization, and P starvation responses with successional time. P limitation inhibited microbial C metabolic potential, as shown by a decrease in CAZy family gene abundance over time, while also stimulating the degradation of lignin compounds may to acquire P resources. Based on metagenomic sequencing, our study provides functional gene-level insights into how P limitation occurring during vegetation succession in subtropical regions inhibits soil microbial C metabolic processes, advancing our understanding of belowground C cycling and microbial metabolic feedback during forest restoration.

## Supplementary Material

Supplementary_information_ycae128

## Data Availability

The raw sequence data generated in this study have been deposited on the NCBI, with the accession number PRJNA1080685.
