# Supplementary material for: Metagenomic insights into inhibition of soil microbial carbon metabolism by phosphorus limitation during vegetation succession
Source: ISME Commun. 2024 Oct 23;4(1):ycae128. doi: 10.1093/ismeco/ycae128 (PMC11538728; doi:10.1093/ismeco/ycae128)
Supplement: Supplementary_information_ycae128 [file supplementary_information_ycae128.docx]

**Supplementary information**

**Title:** Metagenomic insights into inhibition of soil microbial carbon metabolism by phosphorus limitation during vegetation succession


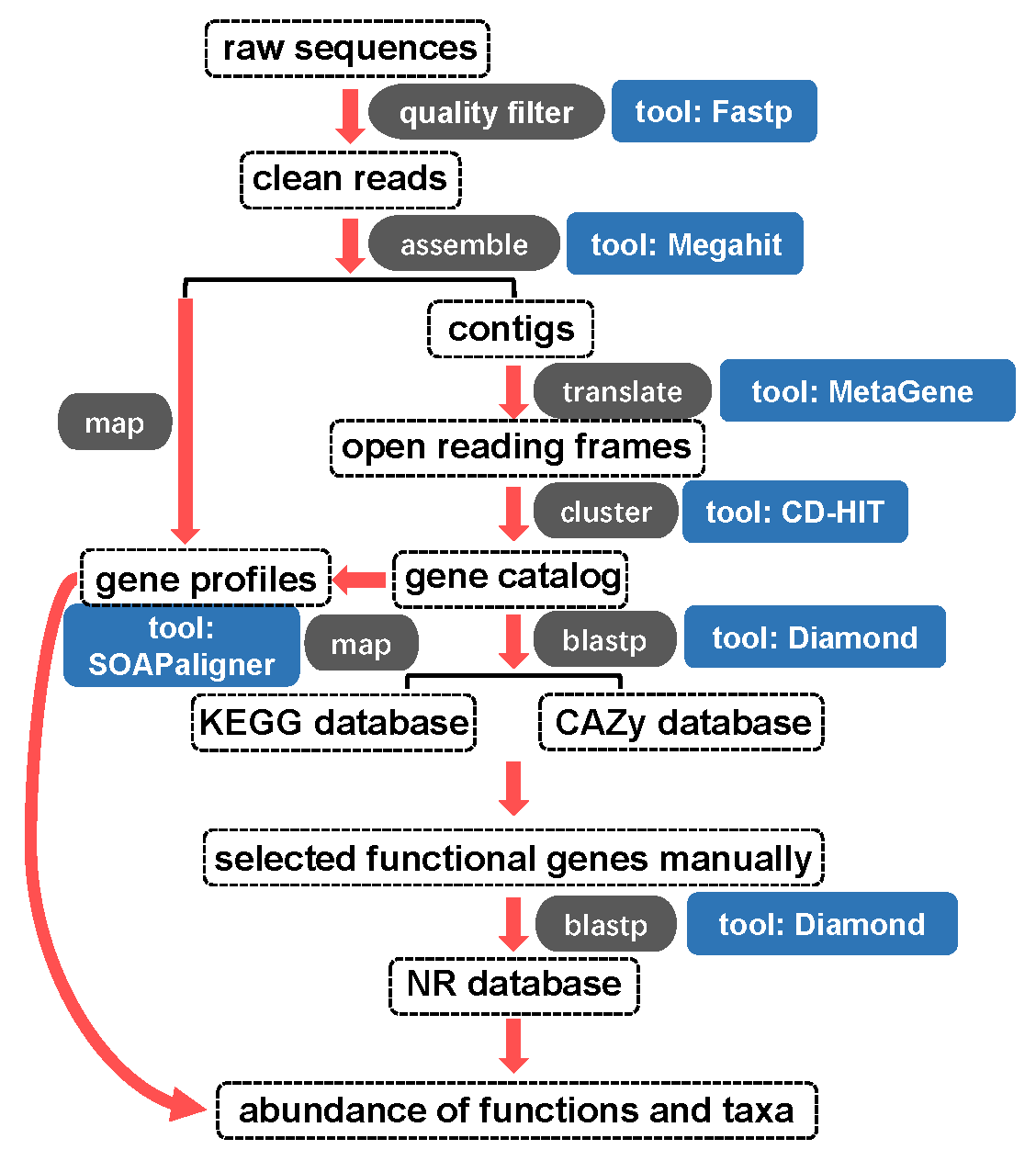


**Fig. S1. The pipeline for analyzing soil metagenomic data.**


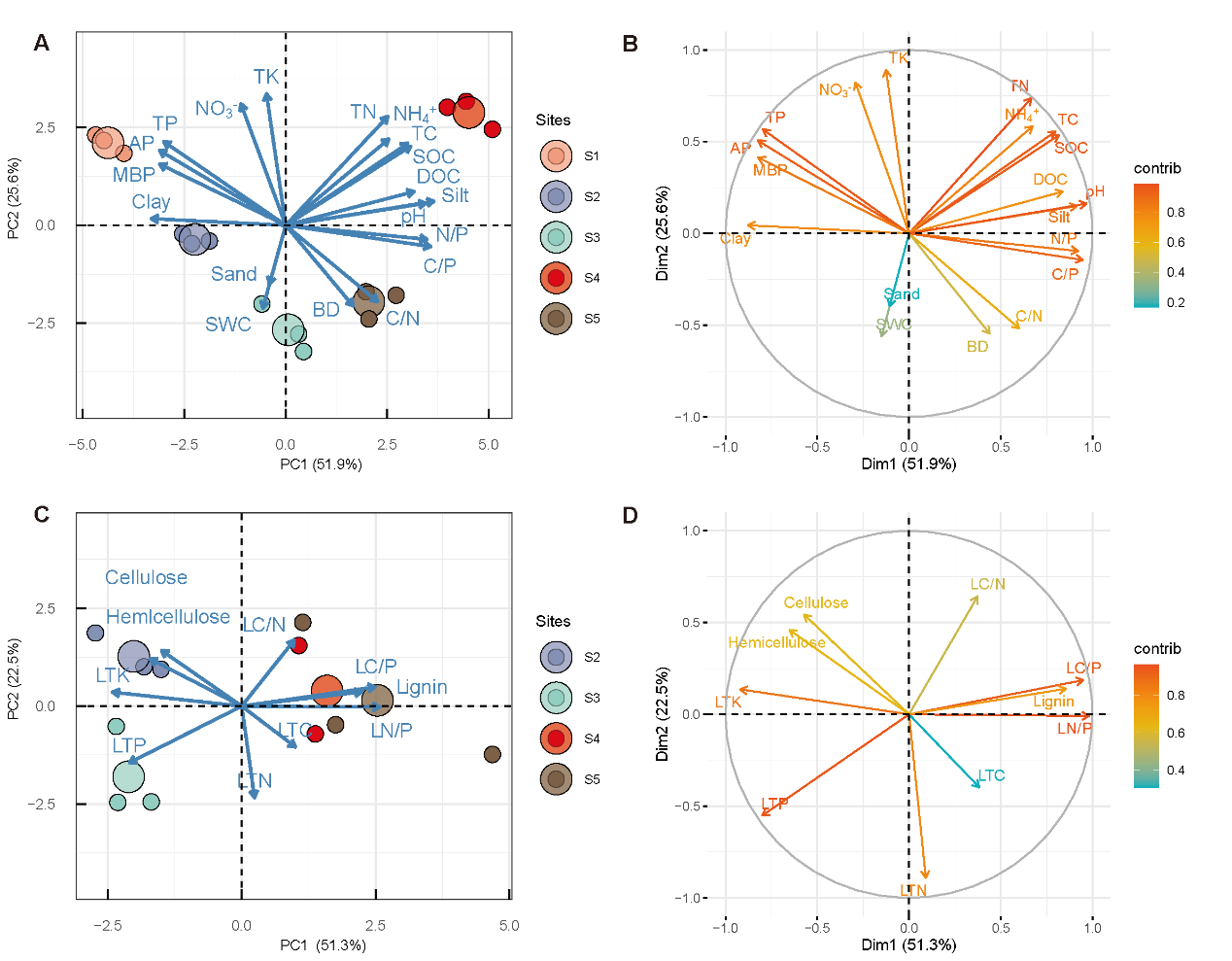


**Fig. S2.** **Principal component analysis (PCA) evaluating variations in soil physicochemical properties (A, B) and litter chemical properties (C, D) with vegetation succession time.** PC1 (explaining 51.9% and 51.3% variations in soil and litter properties, respectively) and PC2 (explaining 25.6% and 22.5% variations in soil and litter properties, respectively) are plotted. Each eigenvector to the variation of principal components is colored according to their contributions to the PC1 and PC2, and the legend titled "contrib" provides the corresponding scale. TP, soil total phosphorus; AP, soil available phosphorus; MBP, microbial biomass phosphorus; TC, soil total carbon; SOC, soil organic carbon; DOC, soil dissolved organic carbon; TN, soil total nitrogen; NH_4_^+^, soil ammonium nitrogen; NO_3_^−^, soil nitrate nitrogen; TK, soil total potassium; SWC, soil water content; BD, soil bulk density; C/N, soil carbon to nitrogen ratio; N/P, soil nitrogen to phosphorus ratio; C/P, soil carbon to phosphorus ratio; LTK, litter total potassium; LTN, litter total nitrogen; LTP, litter total phosphorus; LTC, litter total carbon. The quadratic model was selected based on its lower value of AIC as compared to other models. LC/P, LN/P, and LC/N are denoted as abbreviations for LTC/LTP, LTN/LTP, and LTC/LTN ratio, respectively.


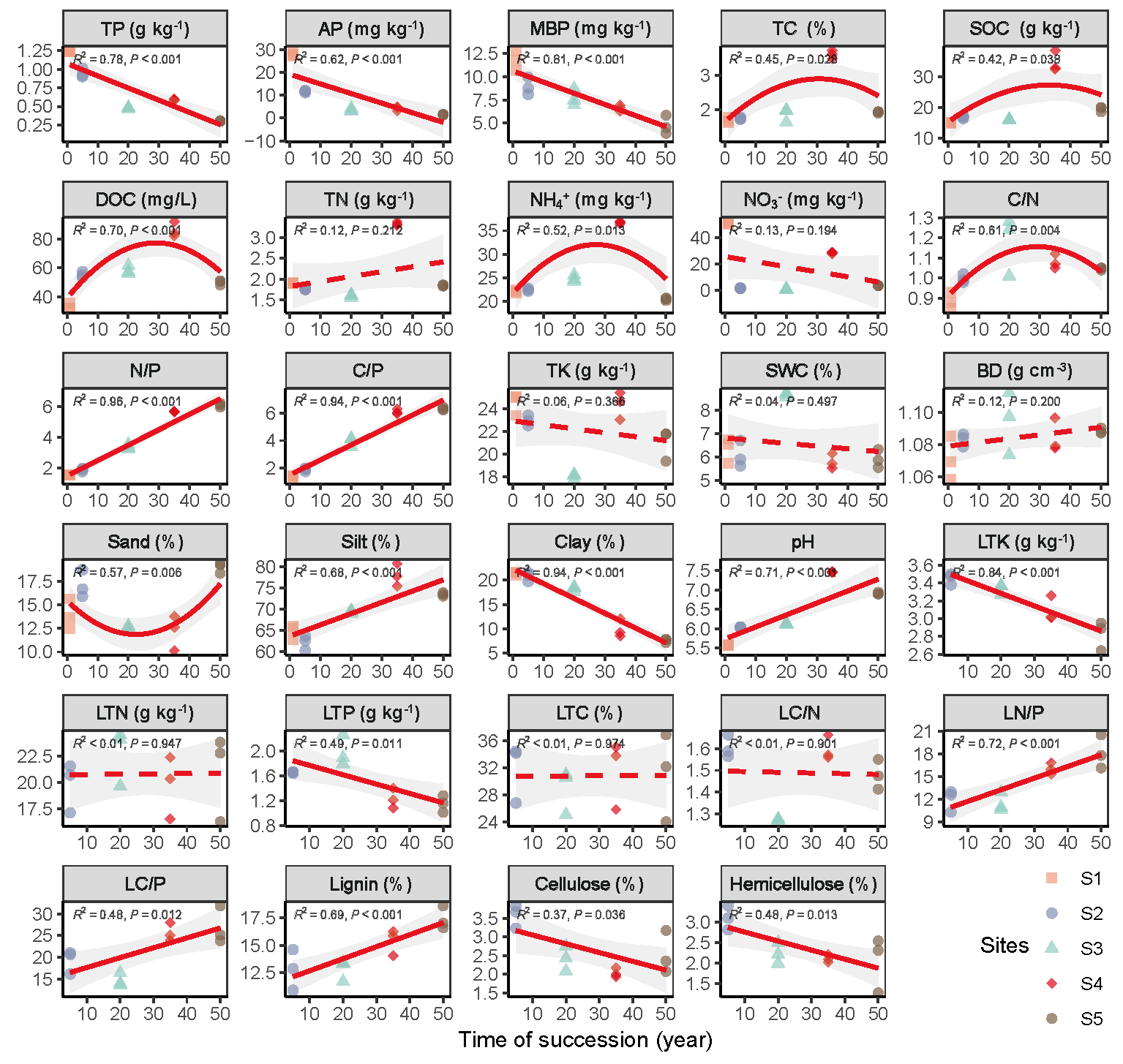


**Fig. S3. The OLS regression between years of succession and the litter-soil physicochemical properties.** The solid lines indicate the fitted model and the gray areas represent the 95% confidence intervals. Linear relationships with no significant correlation use dashed lines. TP, soil total phosphorus; AP, soil available phosphorus; MBP, microbial biomass phosphorus; TC, soil total carbon; SOC, soil organic carbon; DOC, soil dissolved organic carbon; TN, soil total nitrogen; NH_4_^+^, soil ammonium nitrogen; NO_3_^−^, soil nitrate nitrogen; TK, soil total potassium; SWC, soil water content; BD, soil bulk density; C/N, soil carbon to nitrogen ratio; N/P, soil nitrogen to phosphorus ratio; C/P, soil carbon to phosphorus ratio; LTK, litter total potassium; LTN, litter total nitrogen; LTP, litter total phosphorus; LTC, litter total carbon. The quadratic model was selected based on its lower value of AIC as compared to other models. LC/P, LN/P, and LC/N are denoted as abbreviations for LTC/LTP, LTN/LTP, and LTC/LTN ratio, respectively.


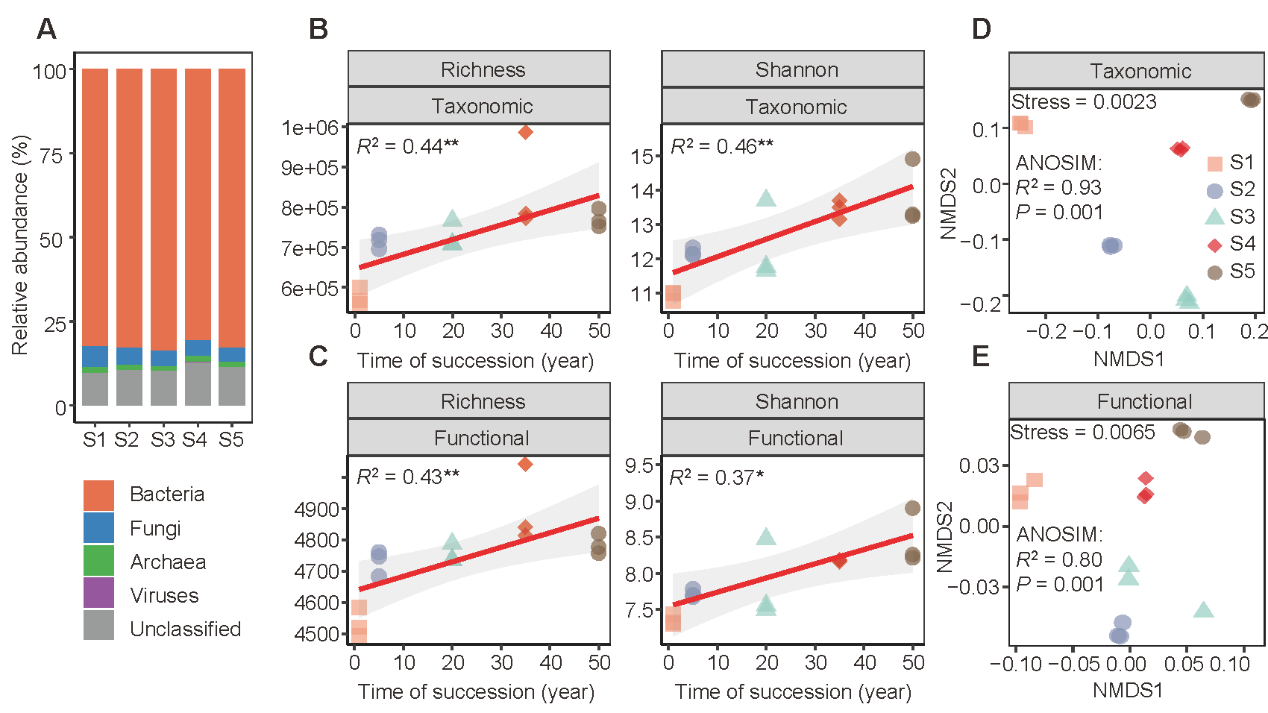


**Fig. S4. The shifts in microbial community compositions, structure, and functional genes derived from metagenomic sequencing data under vegetation succession sequence.** Relative abundance of the bacterial, fungal, and archaeal domains among five different successional stages **(A)**. OLS regression of microbial alpha diversity (for taxonomic and functional) and succession time **(B, C)**. The solid lines indicate the fitted ordinary least-squares model and the gray areas represent the 95% confidence intervals. "*", "**", and "***" indicate the significant levels at *p*-value < 0.05, 0.01, and 0.001, respectively. Non-metric Multidimensional Scaling (NMDS) analysis for microbial communities' taxonomic and functional composition based on Bray–Curtis dissimilarity matrices at all successional stages **(D, E)**. An analysis of similarity (ANOSIM) was used to examine differences in the microbial community and functional profiles for all successional-stage metagenomes.


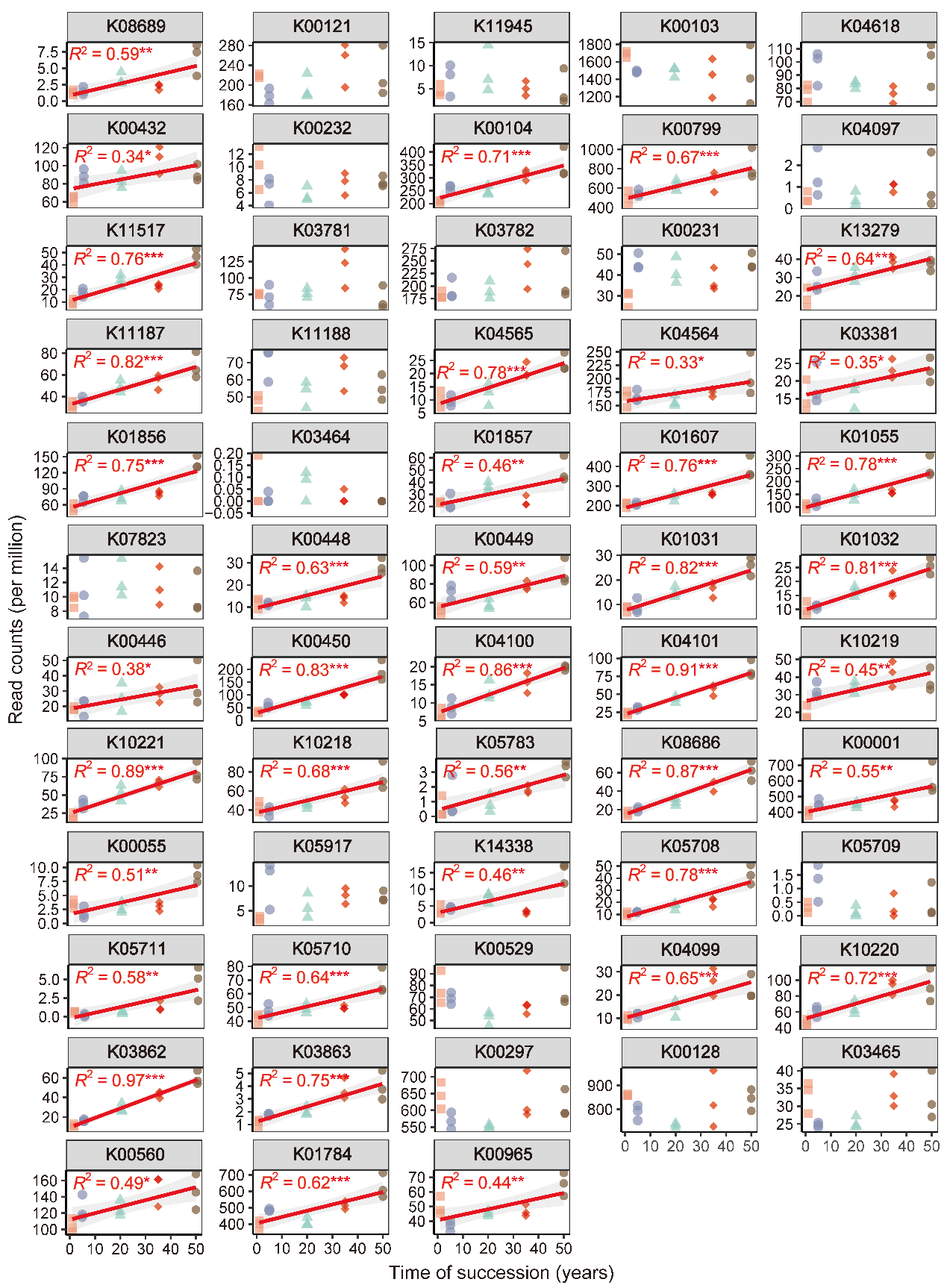


**Fig. S5. Regression of the read numbers (per million) of normalized sequences that were annotated within the 58 enzyme-encoding genes involved in the transformation of lignin and its derived aromatic compounds against years of succession.** The solid line denotes the linear regression and the shaded region denotes the 95% confidence intervals. Only the fitted line when the regression relationship is significant (*P* < 0.05) is shown in each plot. "*", "**", and "***" indicates the significant levels at *P*-value < 0.05, 0.01, and 0.001, respectively.


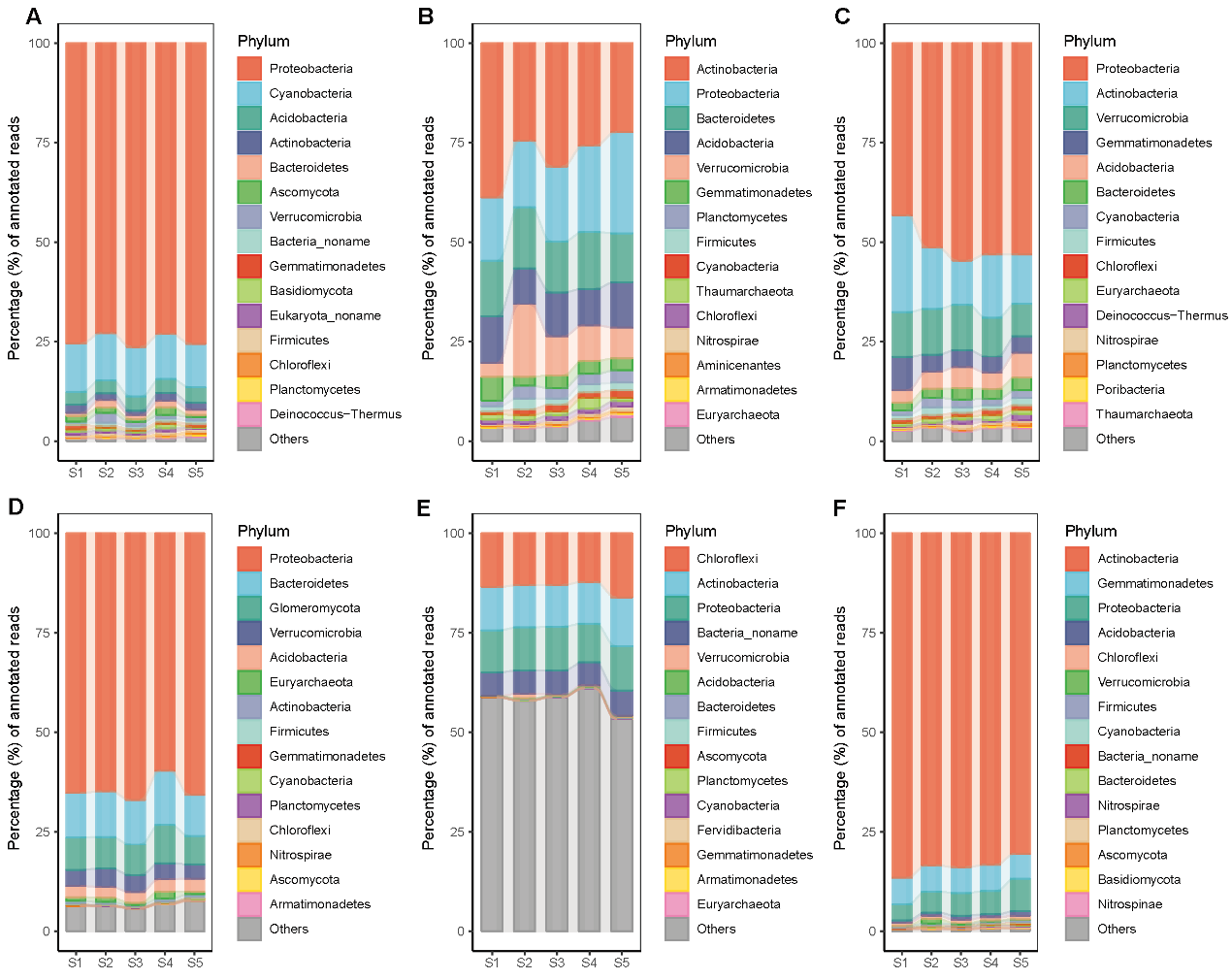


**Fig. S6. The relative abundance of microbial phyla that were invovled in microbial CAZyme genes responsible for the decomposition of plant- and microbial-derived components** **along vegetation succession.** Contribution of microbial (bacterial and fungal) phyla to plant-derived cellulose decomposition **(A)**. Contribution of microbial (bacterial and fungal) phyla to plant-derived hemicellulose decomposition **(B)**. Contribution of microbial (bacterial and fungal) phyla to plant-derived lignin decomposition **(C)**. Contribution of microbial (bacterial and fungal) phyla to fungi-derived chitin decomposition **(D)**. Contribution of microbial (bacterial and fungal) phyla to fungi-derived glucans decomposition **(E)**. Contribution of microbial (bacterial and fungal) phyla to bacteria-derived peptidoglycan decomposition **(F)**. S1, S2, S3, S4, and S5 represent the abbreviations of the five successional stages, as detailed in Table S1.


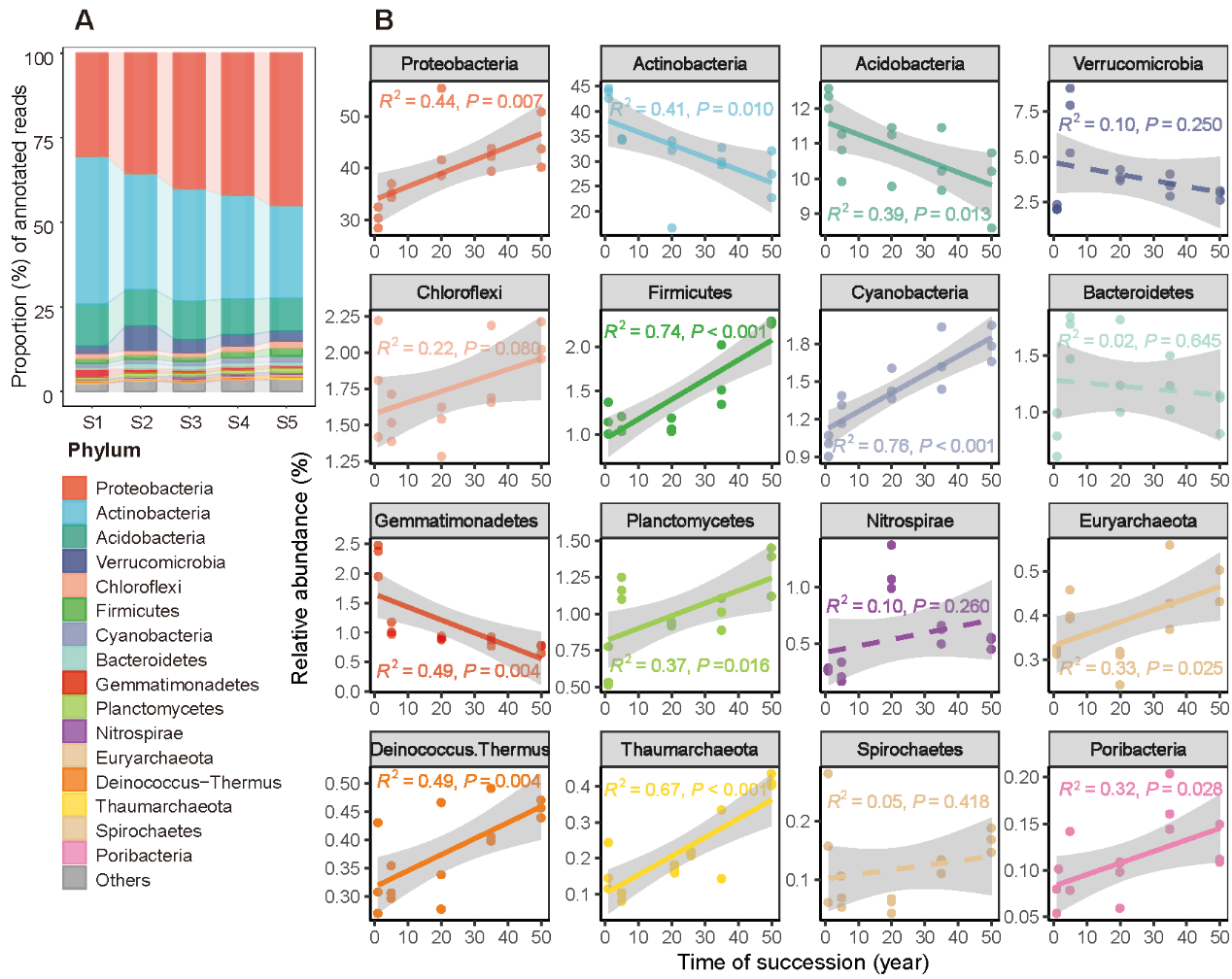


**Fig. S7. The compositions of lignin decomposition-related microbial communities correspond to each successional stage at the phylum level (A). OLS regression analysis shows the relationships between the relative abundance of microbial taxa at the phylum level that were annotated with the 58 enzyme-encoding genes involved in the transformation of lignin and its derived aromatic compounds and years of succession (B).** The solid line denotes the linear regression and the shaded region denotes the 95% confidence intervals, where the dashed line indicates that the regression relationship is not significant.


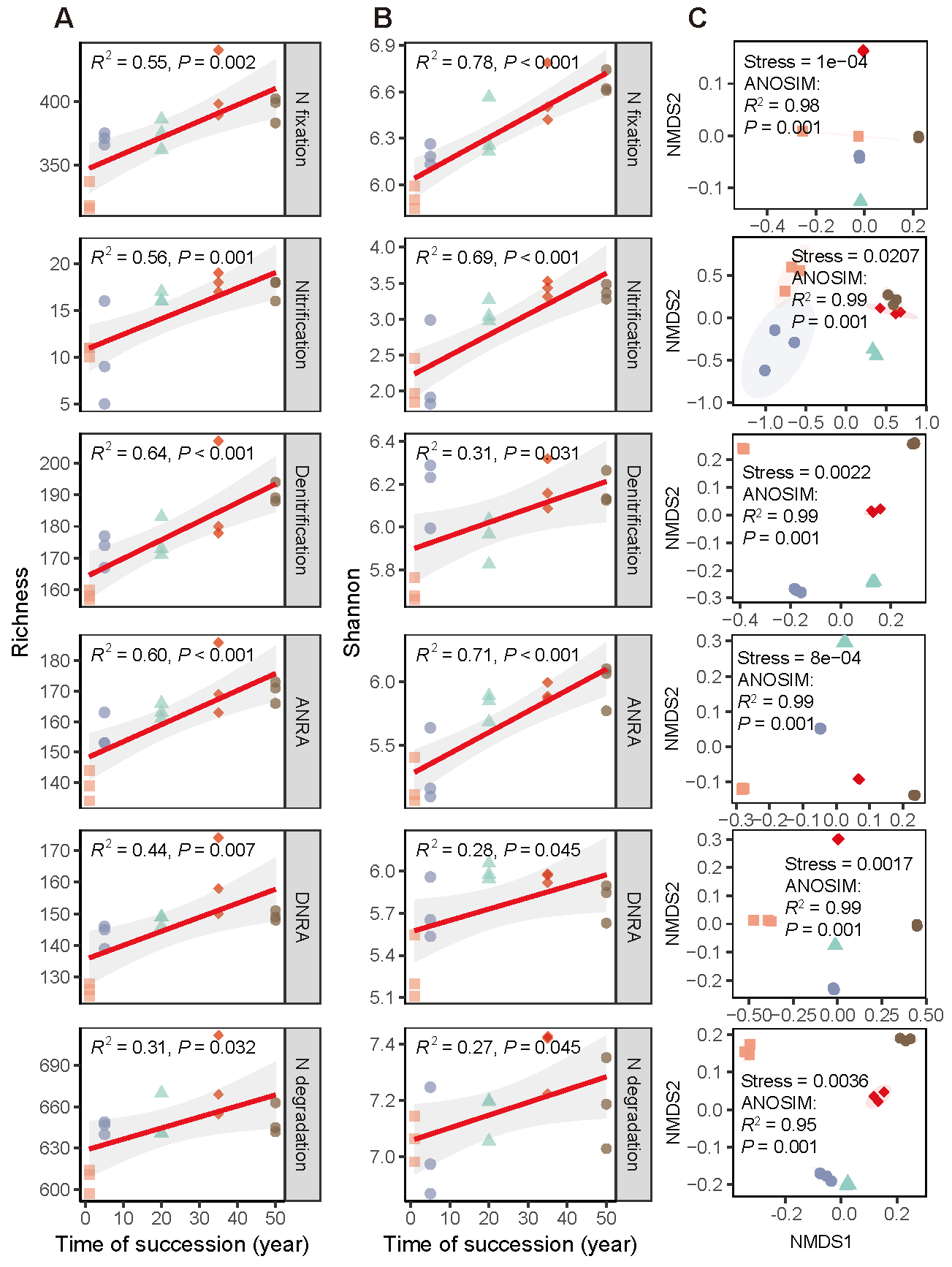


**Fig. S8. Alpha diversity of microbial communities involved in different pathways of the nitrogen cycle with successional time and differences in beta diversity across all successional stages.** OLS regression between alpha diversity of N cycle microbial communities (Richness and Shannon) involved in different N cycle pathways and the time of succession (**A, B**). The solid lines indicate the fitted ordinary least-squares model and the gray areas represent the 95% confidence intervals. Nonmetric multidimensional scaling of (NMDS) of N cycle microbial communities involved in different N cycle pathways based on the Bray-Curtis distance metrics **(C)**. An analysis of similarity (ANOSIM) was used to examine differences in the N cycle microbial communities involved in different N cycle pathways for all successional stages. Oval circles usually represent 95 per cent confidence intervals for samples within a group.


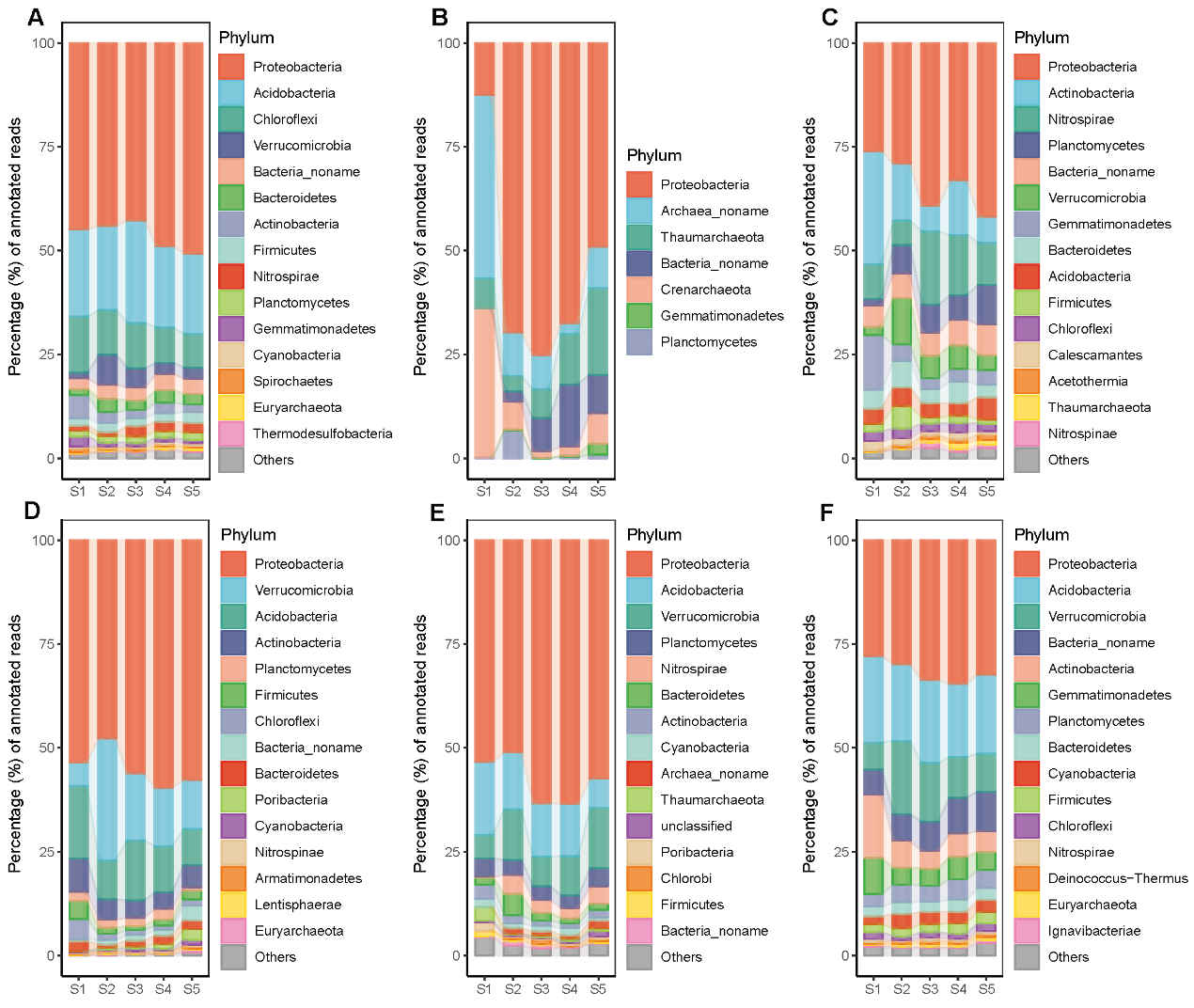


**Fig. S9. Contribution of microbial phyla to microbial KEGG genes in different nitrogen (N) cycle pathways along the vegetation succession.** Contribution of microbial (bacterial and fungal) phyla to N fixation pathways in the nitrogen cycle **(A)**. Contribution of microbial (bacterial and fungal) phyla to nitrification pathways in the N cycle **(B)**. Contribution of microbial (bacterial and fungal) phyla to denitrification pathways in the N cycle **(C)**. Contribution of microbial (bacterial and fungal) phyla to ANRA pathways in the N cycle **(D)**. Contribution of microbial (bacterial and fungal) phyla to DNRA pathways in the N cycle **(E)**. Contribution of microbial (bacterial and fungal) phyla to nitrogen degradation pathways in the N cycle **(F)**.


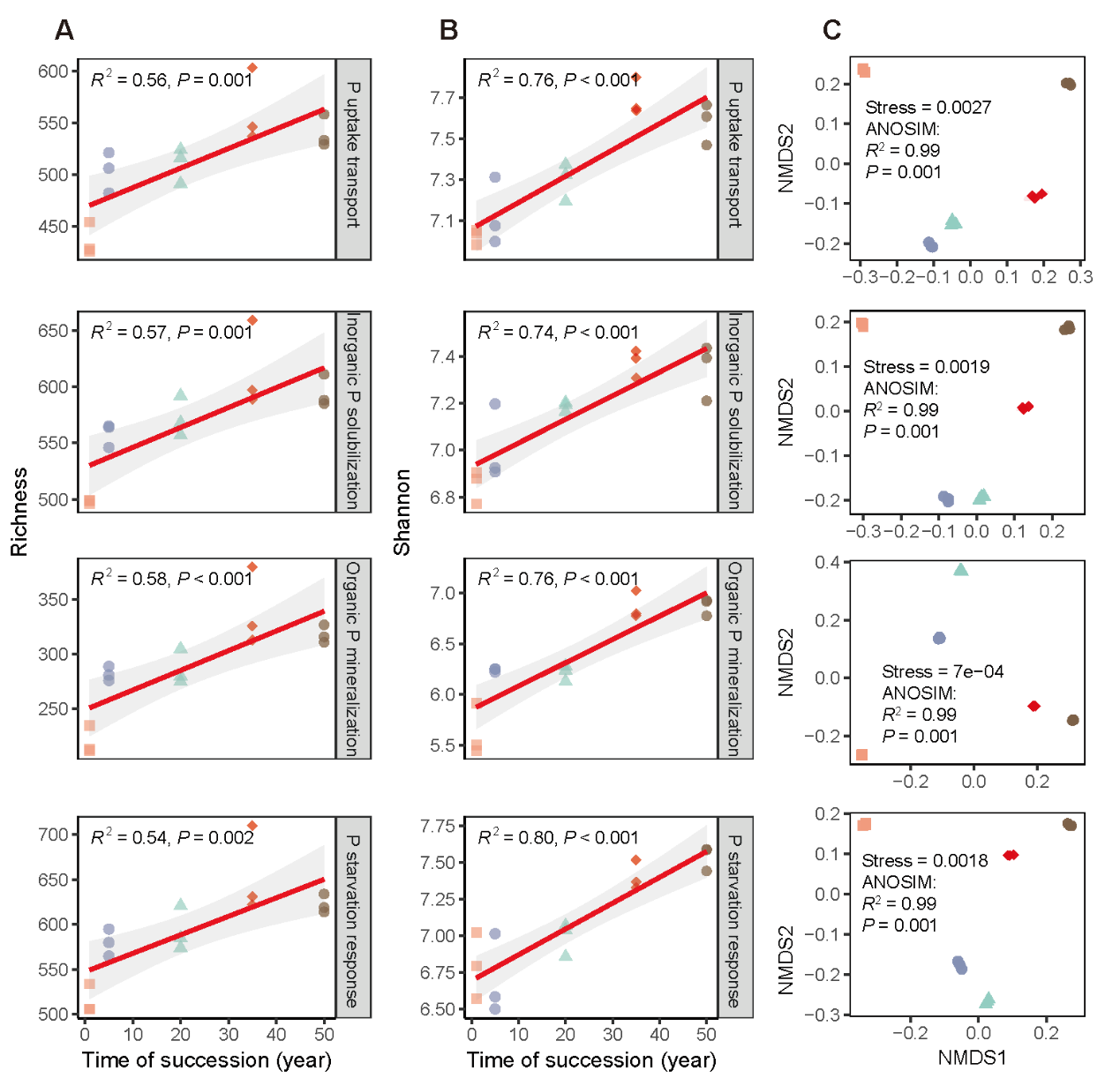


**Fig. S10. Alpha diversity of microbial communities involved in different pathways of the phosphorus (P) cycle with successional time and differences in beta diversity across all successional stages.** The linear regressions between alpha diversity of P-cycle microbial communities (Richness and Shannon) involved in different P-cycle pathways and the time of succession **(A, B)**. The solid lines indicate the fitted ordinary least-squares model and the gray areas represent the 95% confidence intervals. Nonmetric multidimensional scaling of (NMDS) of P-cycle microbial communities involved in different P-cycle pathways based on the Bray-Curtis distance metrics **(C)**. An analysis of similarity (ANOSIM) was used to examine differences in the P-cycle microbial communities involved in different P-cycle pathways for all successional stages. Oval circles usually represent 95 per cent confidence intervals for samples within a group.


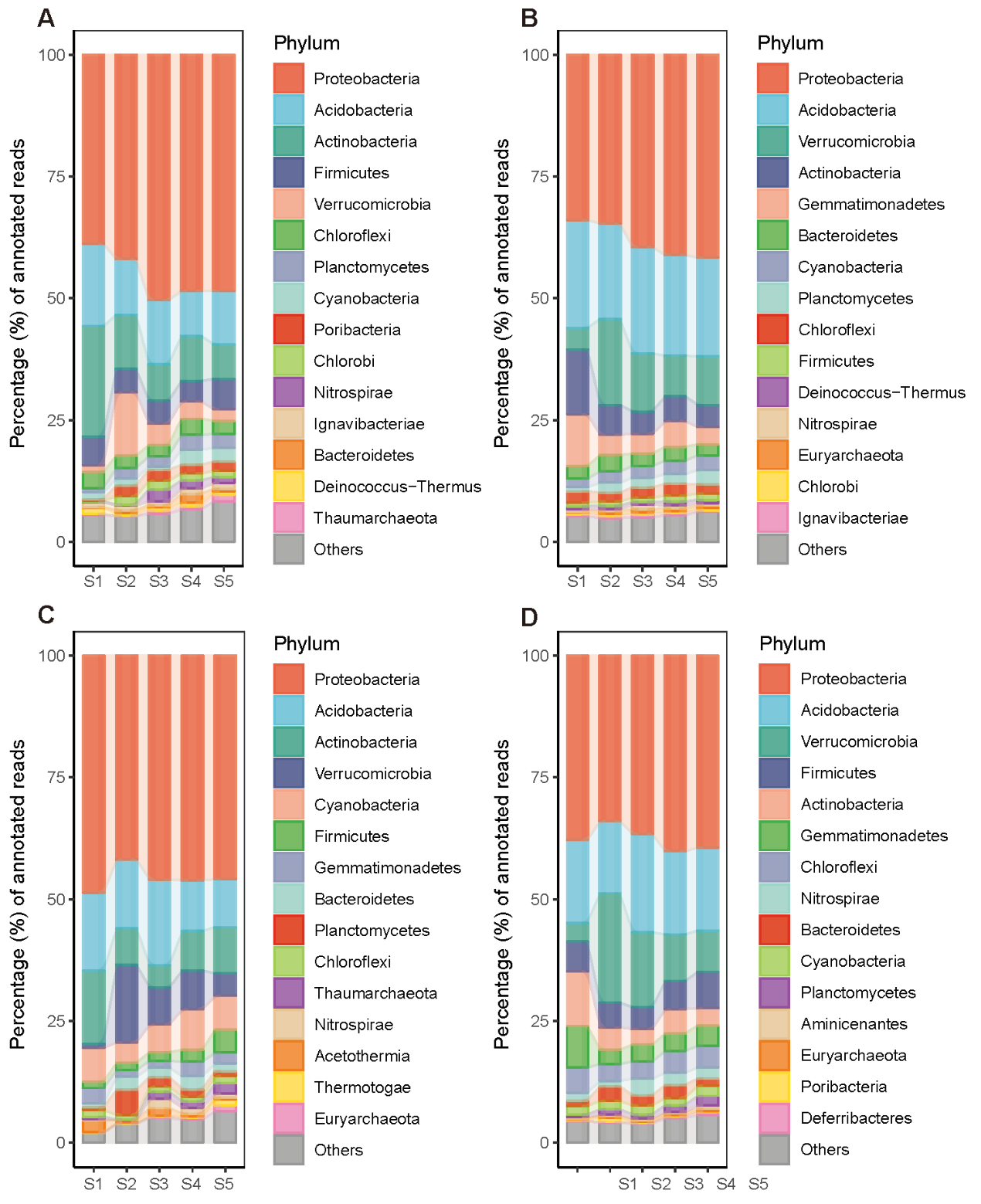


**Fig. S11. Contribution of microbial phyla to microbial KEGG genes in different phosphorus (P) cycle pathways** **along the vegetation succession.** Contribution of microbial (bacterial and fungal) phyla to P uptake and transport **(A)**. Contribution of microbial (bacterial and fungal) phyla to inorganic P solubilization **(B)**. Contribution of microbial (bacterial and fungal) phyla to organic P mineralization **(C)**. Contribution of microbial (bacterial and fungal) phyla to P starvation response regulation **(D)**.


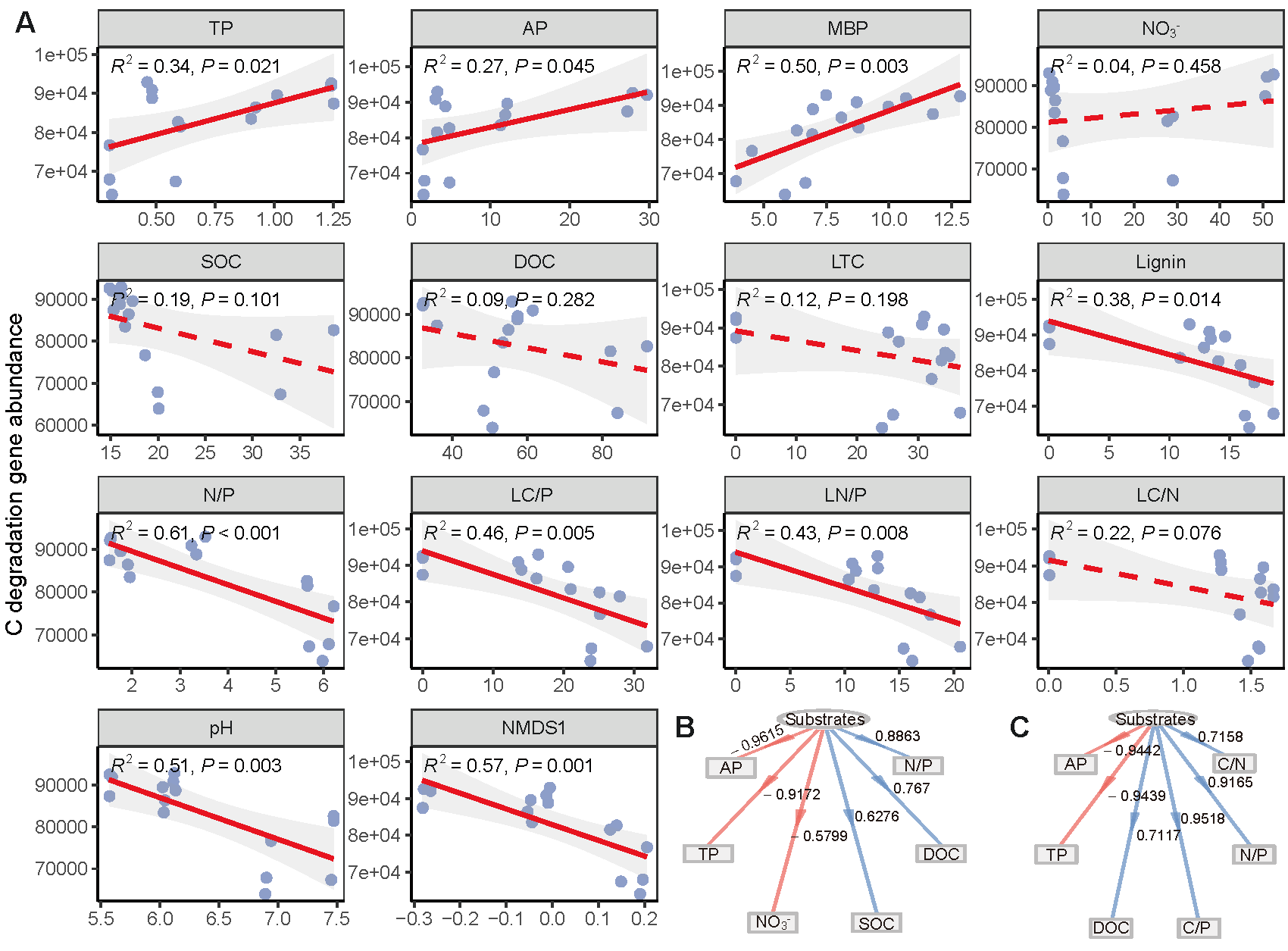


**Fig. S12. Linear regression analysis between the abundance of carbon degradation genes and key environmental driver variables.** The solid lines indicate the fitted ordinary least-squares model and the gray areas represent the 95% confidence intervals **(A)**. The solid line denotes the linear regression and the shaded region denotes the 95% confidence intervals, where the dashed line indicates that the regression relationship is not significant. Statistics of loading values of key substrates as one of the latent variables in Partial Least Squares Path Modelling (PLS-PM) of the response mechanisms of the driver observable variables, i.e., C-degradation genes (**B**) and P-cycling genes (**C**). Loading values reflect the explanatory power of observed variables on latent variables, with higher loading values (greater than 0.7) indicating that the observed variable is a reliable indicator of the latent variable, thereby contributing to the robustness and validity of the model. TP, soil total phosphorus; AP, soil available phosphorus; MBP, microbial biomass phosphorus; NO_3_^−^, soil nitrate nitrogen; SOC, soil organic carbon; DOC, soil dissolved organic carbon; LTC, litter total carbon; N/P, soil nitrogen to phosphorus ratio; C/P, soil carbon to phosphorus ratio; LTN, litter total nitrogen; LTP, litter total phosphorus; The quadratic model was selected based on its lower value of AIC as compared to other models. LC/P, LN/P, and LC/N are denoted as abbreviations for LTC/LTP, LTN/LTP, and LTC/LTN ratio, respectively.

**Table S1. Geographical features and plant characteristics** **at different successional stages.** Except for the differences in vegetation composition at different successional stages, the selected sites had similar geographical characteristics, including elevations, slope gradients, and parent soil material.

| Sites | Stand age (years) | Geographical coordinates | Elevation (m) | Slope gradient (°) | Coverage (%) | Vegetation features | Dominant vegetation species |
| --- | --- | --- | --- | --- | --- | --- | --- |
| S1 | 1 | 29°47′15.72″N, 106°26′35.16″E | 588.1 | ~5 | <30 | The early stage of uncovering: a mix of annual and perennial herbs dominate. | *Alternanthera philoxeroides* (Mart.) Griseb., *Oxaliscorniculata* L., *Stellaria media* (L.) Cyr., *Dendranthema indicum* (L.) Des Moul. |
| S2 | 5 | 29°47′15.74″N, 106°26′34.80″E | 583.6 | ~8 | ~55 | The abandoned land with a natural recovery time of 5 to 8 years is mostly covered with perennial herbaceous plants. | *Saccharum arundinaceum* Retz., *Imperata cylindrica* (L.) Beauv., *Artemisia annua,* *Carex brunnea* Thunb. |
| S3 | 20 | 29°47′4.56″N, 106°26′41.28″E | 563.4 | 10~15 | ~60 | The arboreal layer is not developed and the shrub layer is dominant. | *Viburnum chinshanense* Graebn., *Pyracantha fortuneana* (Maxim.) Li, *Vitex negundo* L., *Zanthoxylum armatum* DC., *Alchornea davidii* Franch., *Myrsine africana* Linn. |
| S4 | 35 | 29°46′48.36″N, 106°26′28.68″E | 578.1 | 12~20 | ~70 | The arboreal layer is not well developed, but the main species composition of the shrub layer is seedlings of broad-leaved tree species. | *Broussonetia papyrifera* (Linn.) L'Hér. Ex Vent., *Celtis sinensis* Pers., *Sapium sebiferum* (L.) Roxb., *Ligustrum lucidum* Ait., *Toona sinensis* (A. Juss.) Roem. *Viburnum chinshanense* Graebn., *Pyracantha fortuneana* (Maxim.) Li, *Elaeagnus pungens* Thunb. |
| S5 | 50 | 29°46′42.61″N, 106°26′22.92″E | 574.6 | 12~20 | ~80 | Dominated by subtropical evergreen broad-leaved forests. | *Cupressus funebris* Endl., *Cinnamomum camphora* (L.) Presl., *Cinnamomum bodinieri*, *Lindera glauca* (Sieb. et Zucc.) Bl, *Sapium sebiferum* (L.) Roxb. |

**Table S2. Basic information of metagenomic sequencing.**

| Sample | Raw reads | Clean reads | Clean%^a^ | Contigs | N50^b^ | N75^c^ | ORFs^d^ |
| --- | --- | --- | --- | --- | --- | --- | --- |
| S1_1 | 59170766 | 56900604 | 96.16 | 82149 | 825 | 604 | 558642 |
| S1_2 | 65744696 | 63344166 | 96.35 | 94667 | 800 | 601 | 600680 |
| S1_3 | 59710312 | 57213152 | 95.82 | 84339 | 1005 | 648 | 567300 |
| S2_1 | 61938296 | 59978030 | 96.84 | 87306 | 815 | 605 | 718581 |
| S2_2 | 63998492 | 62218280 | 97.22 | 99307 | 780 | 598 | 731456 |
| S2_3 | 52838706 | 51099700 | 96.71 | 92384 | 807 | 607 | 695939 |
| S3_1 | 57539658 | 55567910 | 96.57 | 66724 | 896 | 627 | 707118 |
| S3_2 | 62144414 | 60043576 | 96.62 | 87351 | 1060 | 658 | 708127 |
| S3_3 | 82646308 | 80911630 | 97.90 | 263168 | 927 | 638 | 766259 |
| S4_1 | 53436294 | 52177526 | 97.64 | 70292 | 694 | 572 | 986686 |
| S4_2 | 54141826 | 52903938 | 97.71 | 39793 | 647 | 556 | 772625 |
| S4_3 | 60222758 | 58715208 | 97.50 | 18930 | 614 | 544 | 784263 |
| S5_1 | 60506462 | 58705372 | 97.02 | 104307 | 1007 | 657 | 764351 |
| S5_2 | 56587108 | 55262986 | 97.66 | 95970 | 840 | 611 | 752283 |
| S5_3 | 66143226 | 63947282 | 96.68 | 107376 | 871 | 622 | 796246 |

^a^ The percentage of clean read in its corresponding raw read.

^b^ N50 represents the length of the contig overlapping the midpoint of the length-order concatenation of contigs.

^c^ N75 represents the length of the contig overlapping the seventy-five percent of the length-order concatenation of contigs.

^d^ ORFs, Open reading frames.

**Table S3.** Functional classification of glycosyl hydrolases (GH) and auxilliary (AA) encoding the enzymatic activities involved in the plant-and microbial compounds degradation according to CAZy (http://www.CAZy.org).

| **Group** | **Compound** | **CAZy families (GH and AA)** |
| --- | --- | --- |
| Plant biomass | Cellulose | GH1 (β-glucosidase), GH3 (β-glucosidase), GH5 (β-glucosidase/endoglucanase), GH6 (cellobiohydrolase), GH7 (reducing end-acting cellobiohydrolase), GH8 (endoglucanase/endoxylanase), GH9 (endoglucanase), GH12 (endoglucanase), GH45 (endoglucanase), GH48 (reducing end-acting cellobiohydrolase/endoglucanase), GH116 (β-glucosidase), AA9 (lytic polysaccharide monooxygenase), and AA10 (lytic polysaccharide monooxygenase) |
|  | Hemicellulose | GH2 (β-galactosidase/β-glucuronidase), GH10 (endoxylanase), GH11 (endoxylanase), GH26 (endomannanase), GH30 (endoxylanase/β-1,6-glucanase/β-xylosidase), GH36 (α-galactosidase), GH39 (β-xylosidase/α-L-arabinofuranosidase), GH43 (β-xylosidase/endoxylanase), GH44 (xyloglucanase/endoglucanase), GH51 (α-L_x0002_arabinofuranosidase), GH52 (β-xylosidase), GH54 (α-L-arabinofuranosidase), GH62 (α-Larabinofuranosidase) GH67 (xylanα-1,2- glucuronidase), GH74 (xyloglucanase), GH95 (α-L-fucosidase/α-L-galactosidase), GH115 (xylanα-1,2-glucuronidase), GH120 (β-xylosidase) |
|  | Lignin | AA1(laccase), AA2(peroxidase), AA3 (oxidase), AA4 (oxidase), AA5(oxidase), AA6 (1,4-benzoquinone reductase) |
| Fungal biomass | Chitin | GH16 (xyloglucanase/endoglucanase), GH18 (chitinase), GH19 (chitinase), GH20 (N-acetyl β-glucosaminidase), GH72 (β-1,3-glucanosyltransglycosylase) |
|  | Glucans | GH17 (endo-1,3-β-glucanase), GH55 (exo-β-1,3-glucanase/endo-1,3-β-glucanase), GH64 (endo-1,3-β-glucanase), GH81 (endo-1,3-β-glucanase), and GH128 (endo-1,3-β-glucanase) |
| Bacterial biomass | Peptidoglycan | GH22 (lysozyme), GH23 (lysozyme/peptidoglycan lytic transglycosylase), GH24 (lysozyme), GH25 (lysozyme), GH73 (peptidoglycan hydrolase with endo-β-N-acetylglucosaminidase specificity), GH102 (peptidoglycan lytic transglycosylase), GH103 (peptidoglycan lytic transglycosylase), GH104 (peptidoglycan lytic transglycosylase) and GH108 (lysozyme) |

**Table S4.** **The 58 enzyme-encoding genes involved in the transformation of lignin and its derived aromatic compounds.** The table showed the KOs number identifiers, gene or protein name, EC numbers, and putative function or metabolic pathway within the lignin transformation processes.

| **KO ID** | **Gene or Protein ID; Enzyme [EC Number]** | **Function or metabolic pathway within the lignin transformation processes** |
| --- | --- | --- |
| K08689 | *bph*A; biphenyl 2,3-dioxygenase subunit alpha [EC:1.14.12.18] | Catabolism of aromatic compouds |
| K00121 | *frm*A, ADH5, *adh*C; S- (hydroxymethyl) glutathione dehydrogenase/alcohol dehydrogenase [EC:1.1.1.284 1.1.1.1] | Catabolism of aromatic compouds |
| K11945 | *phd*F; extradiol dioxygenase [EC:1.13.11.-] | Catabolism of aromatic compouds |
| K00103 | L-gulonolactone oxidase [EC:1.1.3.8] | Depolymerization of lignin -oxidative stress response |
| K04618 | galactose oxidase [EC:1.1.3.9] | Depolymerization of lignin -oxidative stress response |
| K00432 | glutathione peroxidase [EC:1.11.1.9] | Depolymerization of lignin -oxidative stress response |
| K00232 | ACOX1, ACOX3; acyl-CoA oxidase [EC:1.3.3.6] | Depolymerization of lignin -oxidative stress response |
| K00104 | *glc*D; glycolate oxidase [EC:1.1.3.15] | Depolymerization of lignin -oxidative stress response |
| K00799 | GST, gst; glutathione S-transferase [EC:2.5.1.18] | Depolymerization of lignin -oxidative stress response |
| K04097 | gst; glutathione S-transferase [EC:2.5.1.18] | Depolymerization of lignin -oxidative stress response |
| K11517 | HAO; (S)-2-hydroxy-acid oxidase [EC:1.1.3.15] | Depolymerization of lignin -oxidative stress response |
| K03781 | *kat*E, CAT, catB, srpA; catalase [EC:1.11.1.6] | Depolymerization of lignin -oxidative stress response |
| K03782 | *kat*G; catalase-peroxidase [EC:1.11.1.21] | Depolymerization of lignin -oxidative stress response |
| K00231 | PPOX, *hem*Y; oxygen-dependent protoporphyrinogen oxidase [EC:1.3.3.4] | Depolymerization of lignin -oxidative stress response |
| K13279 | PRDX1; peroxiredoxin 1 [EC:1.11.1.15] | Depolymerization of lignin -oxidative stress response |
| K11187 | PRDX5; peroxiredoxin 5, atypical 2-Cys peroxiredoxin [EC:1.11.1.15] | Depolymerization of lignin -oxidative stress response |
| K11188 | PRDX6; peroxiredoxin 6, 1-Cys peroxiredoxin [EC:1.11.1.7 1.11.1.15 3.1.1.-] | Depolymerization of lignin -oxidative stress response |
| K04565 | SOD1; superoxide dismutase, Cu-Zn family [EC:1.15.1.1] | Depolymerization of lignin -oxidative stress response |
| K04564 | SOD2; superoxide dismutase, Fe-Mn family [EC:1.15.1.1] | Depolymerization of lignin -oxidative stress response |
| K03381 | *cat*A; catechol 1,2-dioxygenase [EC:1.13.11.1] | Fission pathway: beta-ketoadipate pathway: Catechol branch |
| K01856 | *cat*B; muconate cycloisomerase [EC:5.5.1.1] | Fission pathway: beta-ketoadipate pathway: Catechol branch |
| K03464 | *cat*C; muconolactone D-isomerase [EC:5.3.3.4] | Fission pathway: beta-ketoadipate pathway: Catechol branch |
| K01857 | *pca*B; 3-carboxy-cis, cis-muconate cycloisomerase [EC:5.5.1.2] | Fission pathway: beta-ketoadipate pathway: Protocatechuate branch |
| K01607 | *pca*C; 4-carboxymuconolactone decarboxylase [EC:4.1.1.44] | Fission pathway: beta-ketoadipate pathway: Protocatechuate branch |
| K01055 | *pca*D; 3-oxoadipate enol-lactonase [EC:3.1.1.24] | Fission pathway: beta-ketoadipate pathway: Protocatechuate branch |
| K07823 | *pca*F; 3-oxoadipyl-CoA thiolase [EC:2.3.1.174] | Fission pathway: beta-ketoadipate pathway: Protocatechuate branch |
| K00448 | *pca*G; protocatechuate 3,4-dioxygenase, alpha subunit [EC:1.13.11.3] | Fission pathway: beta-ketoadipate pathway: Protocatechuate branch |
| K00449 | *pca*H; protocatechuate 3,4-dioxygenase, beta subunit [EC:1.13.11.3] | Fission pathway: beta-ketoadipate pathway: Protocatechuate branch |
| K01031 | *pca*I; 3-oxoadipate CoA-transferase, alpha subunit [EC:2.8.3.6] | Fission pathway: beta-ketoadipate pathway: Protocatechuate branch |
| K01032 | *pca*J; 3-oxoadipate CoA-transferase, beta subunit [EC:2.8.3.6] | Fission pathway: beta-ketoadipate pathway: Protocatechuate branch |
| K00446 | *dmp*B; catechol 2,3-dioxygenase [EC:1.13.11.2] | Fission pathway: catechol meta-cleavage pathway |
| K00450 | gentisate 1,2-dioxygenase [EC:1.13.11.4] | Fission pathway: gentisate-cleavage pathway |
| K04100 | *lig*A; protocatechuate 4,5-dioxygenase, alpha chain [EC:1.13.11.8] | Fission pathway: protocatechuate 4,5-cleavage pathway |
| K04101 | *lig*B; protocatechuate 4,5-dioxygenase, beta chain [EC:1.13.11.8] | Fission pathway: protocatechuate 4,5-cleavage pathway |
| K10219 | *lig*C; 2-hydroxy-4-carboxymuconate semialdehyde hemiacetal dehydrogenase [EC:1.1.1.312] | Fission pathway: protocatechuate 4,5-cleavage pathway |
| K10221 | *lig*I; 2-pyrone-4,6-dicarboxylate lactonase [EC:3.1.1.57] | Fission pathway: protocatechuate 4,5-cleavage pathway |
| K10218 | *lig*K, *gal*C; 4-hydroxy-4-methyl-2-oxoglutarate aldolase [EC:4.1.3.17] | Fission pathway: protocatechuate 4,5-cleavage pathway |
| K05783 | *ben*D-*xyl*L; dihydroxycyclohexadiene carboxylate dehydrogenase [EC:1.3.1.25 1.3.1.-] | Funneling pathway: benzoic acid |
| K08686 | 2-chlorobenzoate 1,2-dioxygenase [EC:1.14.12.13] | Funneling pathway: benzoic acid |
| K00001 | *adh*; alcohol dehydrogenase [EC:1.1.1.1] | Probably involved in Funneling pathway: coniferyl alcohol |
| K00055 | aryl-alcohol dehydrogenase [EC:1.1.1.90] | Probably involved in Funneling pathway: coniferyl alcohol |
| K05917 | CYP51; cytochrome P450, family 51 (sterol 14-demethylase) [EC:1.14.13.70] | Funneling pathway: guaiacol |
| K14338 | *cyp*D_E, CYP102A2_3; cytochrome P450 / NADPH-cytochrome P450 reductase [EC:1.14.14.1 1.6.2.4] | Funneling pathway: guaiacol |
| K05708 | *hca*A1, *hca*E; 3-phenylpropionate/cinnamic acid dioxygenase subunit alpha [EC:1.14.12.19] | Phenylpropionic acid metabolism |
| K05709 | *hca*A2, *hca*F; 3-phenylpropionate/cinnamic acid dioxygenase subunit beta [EC:1.14.12.19] | Phenylpropionic acid metabolism |
| K05711 | *hca*B; 2,3-dihydroxy-2,3-dihydro phenylpropionate dehydrogenase [EC:1.3.1.87] | Phenylpropionic acid metabolism |
| K05710 | *hca*C, *bph*F; dioxygenase ferredoxin subunit | Phenylpropionic acid metabolism |
| K00529 | *hca*D; ferredoxin--NAD+ reductase [EC:1.18.1.3] | Phenylpropionic acid metabolism |
| K04099 | *des*B, *gal*A; gallate dioxygenase [EC:1.13.11.57] | Funneling pathway: syringic acid I |
| K10220 | *lig*J; 4-oxalmesaconate hydratase [EC:4.2.1.83] | Funneling pathway: syringic acid I/Fission pathway: protocatechuate 4,5-cleavage pathway |
| K03862 | *van*A; vanillate monooxygenase [EC:1.14.13.82] | Funneling pathway: vanillin I |
| K03863 | *van*B; vanillate monooxygenase [EC:1.14.13.82] | Funneling pathway: vanillin I |
| K00297 | *met*F; methylenetetrahydrofolate reductase (NADPH) [EC:1.5.1.20] | O demethylation/C1 metabolism |
| K00128 | aldehyde dehydrogenase (NAD+) [EC:1.2.1.3] | Probably involved in syringaldehyde catabolism |
| K03465 | *thy*X, thy1; thymidylate synthase (FAD) [EC:2.1.1.148] | Tolerance to lignocellulose-derived inhibitors (e.g. furanic compunds) |
| K00560 | *thy*A; thymidylate synthase [EC:2.1.1.45] | Tolerance to lignocellulose-derived inhibitors (e.g. furanic compunds) |
| K01784 | *gal*E, GALE; UDP-glucose 4-epimerase [EC:5.1.3.2] | Tolerance to lignocellulose-derived inhibitors (e.g. furanic compunds) |
| K00965 | *gal*T, GALT; UDP-glucose--hexose-1-phosphate uridylyltransferase [EC:2.7.7.12] | Transporter of gallate and protocatechuate |

**Table S5.** **Information of microbial functional genes involved in the N cycling processes was identified in this study.** The table showed the KOs number identifiers, gene or protein name, EC numbers, and putative function or metabolic pathway within the N cycling processes.

| **KEGG orthology number** | **Gene name** | **Encoded protein [EC]** |
| --- | --- | --- |
| **Nitrogen fixation** | | |
| K02584 | *nifA* | nif-specific regulatory protein |
| K04487 | *nifS* | Nitrogenase metalloclusters biosynthesis protein [EC:2.8.1.7] |
| K02588 | *nifH* | Nitrogenase subunit *Nif*H (ATPase) [EC:1.18.6.1] |
| **Nitrification** | | |
| K10944 | *amoA* | ammonia monooxygenase subunit A/methane [EC:1.14.99.39,1.14.18.3] |
| K10945 | *amoB* | ammonia monooxygenase subunit B/methane |
| K10946 | *amoC* | ammonia monooxygenase subunit C/methane |
| K10535 | *hao* | hydroxylamine dehydrogenase/Hydroxylamine oxidoreductase [EC:1.7.2.6, 1.7.2.8] |
| **Denitrification** | | |
| K00370 | *narG* | nitrate reductase/nitrite oxidoreductase, alpha subunit [EC:1.7.5.1 1.7.99.-] |
| K00371 | *narH* | nitrate reductase/nitrite oxidoreductase, beta subunit [EC:1.7.5.1 1.7.99.-] |
| K00368 | *nirK* | nitrite reductase (NO-forming) [EC:1.7.2.1] |
| K15864 | *nirS* | nitrite reductase (NO-forming) [EC:1.7.2.1 1.7.99.1] |
| K04561 | *norB* | nitric oxide reductase subunit B [EC:1.7.2.5] |
| K02305 | *norC* | nitric oxide reductase subunit C |
| K00376 | *nosZ* | nitrous-oxide reductase [EC:1.7.2.4] |
| **Assimilatory nitrate reduction (ANRA)** | | |
| K00372 | *nasA* | assimilatory nitrate reductase catalytic subunit [EC:1.7.99.-] |
| K15578 | *nasD* | nitrate/nitrite transport system ATP-binding protein [EC:7.3.2.4] |
| K00366 | *nirA* | ferredoxin-nitrite reductase [EC:1.7.7.1] |
| K00367 | *narB* | nitrate reductase [EC:1.7.7.2] |
| K00360 | *nasB* | assimilatory nitrate reductase electron transfer subunit [EC:1.7.99.4] |
| **Dissimilatory nitrate reduction (DNRA)** | | |
| K02567 | *napA* | periplasmic nitrate reductase [EC:1.9.6.1] |
| K02568 | *napB* | periplasmic nitrate reductase, electron transfer subunit |
| K02569 | *napC* | periplasmic nitrate reductase, electron transfer subunit |
| K03385 | *nrfA* | nitrite reductase [EC:1.7.2.2] |
| K15876 | *nrfH* | nitrite reductase complex |
| K00363 | *nirD* | nitrite reductase (NAD(P)H) [EC:1.7.1.15] |
| K00362 | *nirB* | nitrite reductase [NAD(P)H], large subunit [EC:1.7.1.15] |
| **Nitrogen degradation** | | |
| K01428 | *ureC* | Urease subunit alpha [EC:3.5.1.5] |
| K00261 | *gdhA* | glutamate dehydrogenase [EC:1.4.1.3] |
| K00117 | *gdhB* | glucose/sorbosone dehydrogenase [EC:1.1.5.2] |
| K00284 | *gltB* | Glutamate synthase [NADPH] large chain (NADPH-GOGAT) [EC:1.4.7.1] |
| K00266 | *gltD* | Glutamate synthase [NADPH] small chain [EC:1.4.1.13 1.4.1.14] |
| K01915 | *glnA* | Glutamine synthetase, type I, N-terminal (Glutamate--ammonia ligase I) (GSI) [EC:6.3.1.2] |

**Table S6.** **All investigated genes related to soil P cycling with their functional descriptions and KO numbers.** Genes not retrieved in this study are marked in red.

| **Gene** | **Description** | **KEGG orthology** |
| --- | --- | --- |
| ***Genes coding for P-uptake and transport*** | | |
| *pstA* | phosphate-specific transport system subunit PstA | K02038 |
| *pstB* | phosphate-specific transport system subunit *PstB* | K02036 |
| *pstC* | phosphate-specific transport system subunit *PstC* | K02037 |
| *pstS* | phosphate-specific transport system subunit *PstS* | K02040 |
| *pit* | phosphate inorganic transporter | K03306 |
| *phnC* | phosphonate transporter subunit *PhnC* | K02041 |
| *phnD* | phosphonate transporter subunit *PhnD* | K02044 |
| *phnE* | phosphonate transporter subunit *PhnE* | K02042 |
| *ugpA* | glycerol-3-phosphate transporter subunit *UgpA* | K05814 |
| *ugpB* | glycerol-3-phosphate transporter subunit *UgpB* | K05813 |
| *ugpC* | glycerol-3-phosphate transporter subunit *UgpC* | K05816 |
| *ugpE* | glycerol-3-phosphate transporter subunit *UgpE* | K05815 |
| ***Genes coding for inorganic P-solubilization*** | | |
| *ppa* | inorganic pyrophosphatase | K01507 |
| *ppk1* | polyphosphate kinase | K00937 |
| *ppx* | exopolyphosphatase | K01524 |
| *gcd* | quinoprotein glucose dehydrogenase | K00117 |
| *pqqB* | pyrroloquinoline quinone biosynthesis protein B | K06136 |
| *pqqC* | pyrroloquinoline-quinone synthase | K06137 |
| ***Genes coding for organic P-mineralization*** | | |
| *phnA* | phosphonoacetate hydrolase | K19670 |
| *phnP* | phosphoribosyl 1,2-cyclic phosphate phosphodiesterase | K06167 |
| *phnW* | 2-aminoethylphosphonate-pyruvate transaminase | K03430 |
| *opd* | phosphotriesterase | K07048 |
| *ugpQ* | glycerophosphoryl diester phosphodiesterase | K01126 |
| *phoA* | alkaline phosphatase (*PhoA*) | K01077 |
| *phoD* | alkaline phosphatase (*PhoD*) | K01113 |
| *phoN* | acid phosphatase (class A) | K09474 |
| ***Genes coding for P-starvation response regulation*** | | |
| *phoB* | two-component system, OmpR family, phosphate regulon response regulator *PhoB* | K07657 |
| *phoP* | two-component system, OmpR family, alkaline phosphatase synthesis response regulator *PhoP* | K07658 |
| *phoR* | two-component system, OmpR family, phosphate regulon sensor histidine kinase *PhoR* | K07636 |
| *phoU* | phosphate transport system protein | K02039 |

**Table S7. The relative abundance of microbial community composition at the different vegetation succession stages.** Different letters indicate significant differences (ANOVA, *P* < 0.05, LSD test) among different successional stages. *, *P* < 0.05; **, *P* < 0.01; ***, *P* < 0.001. S1, S2, S3, S4, and S5 represent the abbreviations of the five successional stages, as detailed in Table S1.

|  | S1 | S2 | S3 | S4 | S5 | F | *P* |
| --- | --- | --- | --- | --- | --- | --- | --- |
| Bacteria | 82.41±0.22a | 82.81±0.23a | 83.78±0.23a | 80.73±0.22a | 82.92±0.23a | 46.17 | 1.92e-05 *** |
| Fungi | 5.97±0.01a | 4.87±0.01ab | 4.35±0.01b | 4.35±0.01b | 3.85±0.01b | 42.55 | 2.84e-05 *** |
| Archaea | 2.06±0.01a | 1.91±0.01a | 1.71±0.01a | 2.1±0.01a | 1.83±0.01a | 8.269 | 0.0139 * |
| Viruses | 0.01±0.03ab | 0.02±0.03ab | 0.02±0.03ab | 0.04±0.04a | 0.01±0.03b | 0.016 | 0.903 |
| Unclassified | 9.55±0.002a | 10.4±0.002a | 10.14±0.005a | 12.78±0.01a | 11.38±0.003a | 2.28 | 0.133 |

**Table S8. The abundance of selected microbial CAZymes genes (mean ± SE) encoding the decomposition of the plant-and microbial-derived components along vegetation succession.** Different letters indicate significant differences (ANOVA, *P* < 0.05, LSD test) among different successional stages. *, *P* < 0.05; **, *P* < 0.01; ***, *P* < 0.001.

|  | CAZy families | S1 | S2 | S3 | S4 | S5 | F | *P* |
| --- | --- | --- | --- | --- | --- | --- | --- | --- |
| Cellulose | GH1 | 189.7±3.43a | 178.53±3.61a | 136.37±12.4a | 157.24±7.03a | 159.5±11.69a | 3.822 | 0.044* |
| Cellulose | GH3 | 611.89±26.91a | 749.59±23.35a | 703.76±12.71a | 696.46±12.71a | 716.88±34.92a | 2.871 | 0.087 |
| Cellulose | GH5 | 394.16±30.41a | 527.04±43.64a | 350.69±6.2a | 540.96±47.37a | 335.74±23.67a | 4.773 | 0.024* |
| Cellulose | GH6 | 41.12±6.12a | 49.19±4a | 28.9±4.86b | 51.96±0.55a | 56.66±5.57a | 2.953 | 0.081 |
| Cellulose | GH8 | 21.77±1.93c | 102.4±9.65a | 65.37±1.04ab | 60.93±7.7b | 67.6±1.95ab | 15.34 | 0.0004*** |
| Cellulose | GH9 | 70.86±5.64b | 120.73±5.87a | 85.63±9.34ab | 123.11±5.4a | 82.3±1.23b | 12.32 | 0.001** |
| Cellulose | GH12 | 143.57±16.41a | 133.9±18.9a | 108.53±6.41a | 141.19±9.95a | 108.78±9.48a | 0.965 | 0.472 |
| Cellulose | GH45 | 4.61±1.59a | 5.07±0.29a | 2.42±0.7a | 3.28±0.85a | 3.06±0.42a | 0.873 | 0.516 |
| Cellulose | GH48 | 10.33±1.39b | 19.81±1.56ab | 21.28±0.56ab | 19.68±2.64ab | 26.87±2.15a | 6.565 | 0.009** |
| Cellulose | GH116 | 5.87±0.93a | 7.97±1.59a | 6.35±0.49a | 8.55±1.28a | 9.65±0.61a | 1.239 | 0.361 |
| Cellulose | AA9 | 0.14±0.05b | 0.19±0.02b | 0.78±0.08b | 0.36±0.01b | 5.58±1.25a | 10.51 | 0.001** |
| Cellulose | AA10 | 9259.49±171.37a | 8587.65±161.22ab | 9438.78±271.52a | 7770.21±623.41ab | 6858.36±321.61b | 5.464 | 0.016* |
| Hemicellulose | GH2 | 578.07±15.12b | 979.29±55.82a | 704.78±11.64b | 726.55±18.4b | 695.17±52.53b | 9.977 | 0.002** |
| Hemicellulose | GH10 | 116.14±13.55b | 181.21±10.11a | 126.02±13.23ab | 146.58±5.47ab | 128.28±6.93ab | 4.374 | 0.031* |
| Hemicellulose | GH11 | 767.35±20.26ab | 717.17±18.63ab | 811.38±31.3a | 598.78±65.82ab | 500.03±48.81b | 5.595 | 0.015* |
| Hemicellulose | GH26 | 59.12±7.6a | 89±15.62a | 56.52±3.51a | 112.13±11.11a | 53.8±3.11a | 4.262 | 0.033* |
| Hemicellulose | GH30 | 33.91±3.59b | 64.31±1.44a | 47.9±2.82ab | 44.92±2.29b | 52.3±3.69ab | 9.839 | 0.002** |
| Hemicellulose | GH36 | 107.19±5.28d | 199.45±2.91bc | 161.63±2.12c | 207.92±9.44b | 264.26±7.25a | 55.49 | 2.42e-06*** |
| Hemicellulose | GH39 | 112.67±8.91b | 218.36±10.85a | 141.61±11.19b | 137.45±9.8b | 140.51±4.18b | 13.02 | 0.0008*** |
| Hemicellulose | GH43 | 277.37±12.51b | 556.49±47.02a | 342.22±22.77b | 379.86±12.15b | 426.54±14.28ab | 10.9 | 0.002** |
| Hemicellulose | GH44 | 14.69±0.33c | 34.03±1.29a | 22.29±4.4bc | 26.27±1.43ab | 22.73±0.59bc | 11.5 | 0.001** |
| Hemicellulose | GH51 | 95.09±4.19c | 163.2±3.84a | 125.7±4.02bc | 133.6±5.74ab | 118.28±5.1bc | 18.67 | 0.0002*** |
| Hemicellulose | GH54 | 43.99±2.98a | 53.26±3.85a | 41.26±12.26a | 26.03±2.56a | 34.52±6.25a | 2.3 | 0.138 |
| Hemicellulose | GH62 | 5.62±0.55b | 19.18±1.4a | 17.02±1.07a | 17.18±2.53a | 11.53±0.35ab | 9.601 | 0.002** |
| Hemicellulose | GH67 | 30.23±1.29a | 26.15±0.86ab | 29.71±2.07ab | 29±0.39ab | 20.36±2.43b | 4.641 | 0.026* |
| Hemicellulose | GH74 | 253.37±13.46ab | 190.11±5.1b | 223.91±2.32ab | 207.37±13.85ab | 267.19±12.04a | 5.559 | 0.016* |
| Hemicellulose | GH95 | 64.71±5.17c | 118.69±3.58a | 88.8±0.84bc | 89.24±2.47b | 105.13±3.08ab | 22.07 | 0.0001*** |
| Hemicellulose | GH115 | 14.51±0.64b | 48.14±6.14a | 27.62±3.13ab | 22.26±1.76b | 17.32±0.14b | 11.74 | 0.001** |
| Hemicellulose | GH120 | 4.68±0.31a | 1.58±0.13b | 2.96±1.15ab | 1.63±0.34b | 1.9±0.21ab | 5.998 | 0.012* |
| Lignin | AA1 | 91.21±3.86b | 78.35±2.69b | 81.66±0.83b | 102.88±3.02ab | 127.95±10.79a | 7.751 | 0.005** |
| Lignin | AA2 | 178.55±3.43a | 189.56±10.47a | 180.23±4.54a | 233.4±18.31a | 212.78±22.36a | 1.637 | 0.247 |
| Lignin | AA3 | 339.92±5.71a | 383.51±14.1a | 372.21±11.77a | 403.68±24.15a | 463.31±45.88a | 2.076 | 0.167 |
| Lignin | AA4 | 54.95±1.3c | 81.14±4.71bc | 91.83±0.88ab | 91.03±1.12ab | 110.73±8.15a | 13.44 | 0.0008*** |
| Lignin | AA5 | 81.35±3.14b | 129.5±10.42a | 106.55±2.93ab | 95.04±5.76ab | 132.61±7.06a | 7.09 | 0.007** |
| Lignin | AA6 | 9.74±1.49c | 16.92±1.43bc | 25.61±1.77ab | 25.37±0.64b | 38.6±3.21a | 21.32 | 0.0001*** |
| Chitin | GH16 | 5930.86±127.84a | 5691.39±245.17a | 5628.41±114.49a | 5577.17±472.56a | 4916.75±279.68a | 1.115 | 0.407 |
| Chitin | GH18 | 555.17±30.69a | 679.99±18.59a | 550.48±4.46a | 633.94±11.31a | 691.74±30.12a | 5.183 | 0.019* |
| Chitin | GH19 | 48668.1±1194.18a | 45289.76±1012.44a | 49773.37±1170.2a | 37924.94±2276.62ab | 34444.45±2172.92b | 9.639 | 0.003** |
| Chitin | GH20 | 133.1±6.75b | 160.6±4.69b | 140.31±3.45b | 146.32±5.52b | 203.08±7.07a | 14.7 | 0.0006*** |
| Chitin | GH72 | 156.81±7.01b | 183.17±3.6ab | 183.9±0.44ab | 220.83±15.74a | 193.93±9.8ab | 3.917 | 0.041* |
| Glucans | GH17 | 6566.6±102.85a | 6071.2±50.6a | 6396.3±24.17a | 6249.83±368.77a | 4221.08±910.56a | 2.739 | 0.096 |
| Glucans | GH55 | 1214.33±15.89a | 1148.91±15.95a | 1152.53±9.62a | 1094.03±67.94a | 994.79±31.05a | 3.278 | 0.064 |
| Glucans | GH64 | 9.67±2.02b | 107.54±20.13a | 41.28±1.14ab | 28.21±6.18b | 16.01±2.15b | 10.26 | 0.002** |
| Glucans | GH81 | 3308.35±26.14a | 3022.17±28.95a | 3207.95±52.57a | 2790.07±252.06a | 2572.87±156.03a | 2.854 | 0.088 |
| Glucans | GH128 | 32.65±3.3c | 104.94±8.87a | 51.29±3.53bc | 64.08±4.02bc | 79.24±4.78b | 16.44 | 0.0004*** |
| Peptidoglycan | GH22 | 639.07±13.59a | 574.47±9.34a | 570.33±5.12a | 576.94±31.5a | 533.51±36.51a | 1.654 | 0.244 |
| Peptidoglycan | GH23 | 9136.03±195.2a | 8068.71±96.26a | 8076.61±58.88a | 8100.6±570.59a | 7873.42±424.04a | 1.355 | 0.323 |
| Peptidoglycan | GH24 | 26.45±3.24b | 54.1±5.96a | 32.33±4.19ab | 27.15±2.22b | 33.46±3.53ab | 5.334 | 0.018* |
| Peptidoglycan | GH25 | 45.37±4.83ab | 68.95±3.76a | 37.55±5.72b | 55.26±3.54ab | 55.5±3.75ab | 4.594 | 0.027* |
| Peptidoglycan | GH73 | 55.38±3.4b | 97.98±7.7a | 93.47±8.9ab | 80.21±4.23ab | 97.02±7.16a | 5.389 | 0.017* |
| Peptidoglycan | GH102 | 17.11±3.35a | 18.56±1.26a | 17.52±1.84a | 22.23±1.32a | 31.1±3.6a | 3.392 | 0.059 |
| Peptidoglycan | GH103 | 31.78±2.52b | 45.74±4.97ab | 56.31±7.95ab | 49.18±2.97ab | 73.1±7.36a | 5.575 | 0.015* |
| Peptidoglycan | GH104 | 0.29±0.05c | 2.17±0.37c | 0.78±0.29bc | 3.91±0.33a | 3.17±0.84b | 6.692 | 0.009** |
| Peptidoglycan | GH108 | 7.12±0.78c | 11.76±0.71bc | 12.43±1.17bc | 15.98±1.2b | 18.31±0.46a | 16.25 | 0.0004*** |

**Table S9.** **Differences in abundance (mean ± SE) of selected microbial nitrogen (N) cycling genes with successional time.** Different letters indicate significant differences (ANOVA, *P* < 0.05, LSD test) among different successional stages. *, *P* < 0.05; **, *P* < 0.01; ***, *P* < 0.001.

| KEGG number | Gene name | S1 | S2 | S3 | S4 | S5 | F | *P* |
| --- | --- | --- | --- | --- | --- | --- | --- | --- |
| K02584 | *nifA* | 377.75c±8.54 | 451.55bc±11.35 | 443.88bc±30.97 | 592.92ab±14.08 | 663.81a±49.35 | 46.17 | 1.92e-05 *** |
| K04487 | *nifS* | 315.61c±3.36 | 361.61bc±3.86 | 338.22bc±7.03 | 411.23ab±16.5 | 443.57a±11.49 | 42.55 | 2.84e-05 *** |
| K02588 | *nifH* | 6.62ab±0.22 | 4.77b±0.52 | 9.63ab±2.04 | 5.31b±0.14 | 18.91a±2.05 | 8.269 | 0.0139 * |
| K10944 | *amoA* | 5.17a±0.99 | 0.68a±0.1 | 5.43a±0.46 | 2.87a±0.46 | 4.32a±0.52 | 0.016 | 0.903 |
| K10945 | *amoB* | 4.16a±0.95 | 0.43a±0.09 | 2.71a±0.23 | 2.65a±0.44 | 2.92a±0.06 | 0.008 | 0.932 |
| K10946 | *amoC* | 0.9b±0.01 | 1.17b±0.9 | 23.4a±1.43 | 12.18ab±1.46 | 12.82ab±1.45 | 7.824 | 0.0161 * |
| K10535 | *hao* | 1.37c±0.09 | 2.88bc±0.46 | 5abc±0.37 | 6.83a±0.45 | 5.59ab±0.54 | 30.92 | 0.0001 *** |
| K00370 | *narG* | 73.57a±2.21 | 49.87a±1.91 | 61.53a±5.54 | 53.16a±1.06 | 98.81a±8.53 | 2.229 | 0.161 |
| K00371 | *narH* | 48.84a±0.63 | 36.61a±1.25 | 29.65a±1.72 | 28.5a±1.42 | 53.02a±6.84 | 0 | 0.991 |
| K00368 | *nirK* | 57.88b±6.52 | 68.4b±5.42 | 84.21b±1.86 | 84.3b±3.19 | 139.68a±4.36 | 36.24 | 6.03e-05 *** |
| K15864 | *nirS* | 1.43b±0.14 | 1.35b±0.15 | 7.66ab±0.1 | 1.82ab±0.17 | 9.54a±0.61 | 10.65 | 0.00679 ** |
| K04561 | *norB* | 48.3a±2.41 | 52.29a±2.62 | 34.67a±1.52 | 43.11a±0.38 | 47.67a±2.87 | 0.663 | 0.431 |
| K02305 | *norC* | 1.39b±0.23 | 3.82ab±0.2 | 9a±0.27 | 8.63a±1.08 | 8.73a±0.65 | 33.84 | 8.25e-05 *** |
| K00376 | *nosZ* | 60.29a±2.47 | 42.03a±1.77 | 34.35a±3.8 | 40.16a±2.12 | 70.89a±3.38 | 0.486 | 0.499 |
| K00372 | *nasA* | 125.15a±3.15 | 150.76a±2.08 | 135.87a±2.04 | 170.73a±8.06 | 154.62a±11.09 | 6.348 | 0.0269 * |
| K15578 | *nasD* | 29.37b±0.1 | 88.89a±4.65 | 84.26ab±6.86 | 121.58a±8.91 | 124.48a±13.11 | 33.17 | 9.02e-05 *** |
| K00366 | *nirA* | 17.31a±2.58 | 34.96a±1.22 | 28.66a±4.29 | 24.37a±0.18 | 37.61a±3.9 | 3.983 | 0.0692 |
| K00367 | *narB* | 2.18b±0.44 | 2.77b±0.44 | 5.11b±0.16 | 6.79b±1.18 | 14.14a±1.65 | 33.98 | 8.1e-05 *** |
| K00360 | *nasB* | 1.54a±0.43 | 0.68a±0.2 | 0.03a±0.03 | 0.28a±0.14 | 0.24a±0.09 | 8.878 | 0.0115 * |
| K02567 | *napA* | 10.4b±1.71 | 16.83b±1.58 | 23.04ab±2.49 | 40.16a±4.19 | 28.46ab±2.48 | 16.31 | 0.00164 ** |
| K02568 | *napB* | 1.09b±0.17 | 4.67b±1.1 | 7.37ab±0.09 | 7.33b±1.05 | 14.64a±1.25 | 48.22 | 1.55e-05 *** |
| K03385 | *nrfA* | 28.81c±1.56 | 50.54abc±3.88 | 30.74bc±2.53 | 66.05ab±0.51 | 76.51a±4.1 | 28.31 | 0.0001 *** |
| K15876 | *nrfH* | 11.8b±1.39 | 17.07b±2.03 | 13.77b±2.26 | 23.06ab±1.3 | 37.1a±0.96 | 34.63 | 7.42e-05 *** |
| K00363 | *nirD* | 30.55b±2.76 | 52.85ab±4.18 | 41.25ab±0.86 | 51.49ab±1.94 | 59.98a±6.83 | 9.674 | 0.00902 ** |
| K00362 | *nirB* | 59.03a±3.56 | 81.47a±3.78 | 79.65a±5.21 | 134.34a±15.73 | 70.27a±4.01 | 1.843 | 0.2 |
| K01428 | *ureC* | 84.23a±3.32 | 124.96a±7.35 | 132.53a±1.46 | 178.96a±19.4 | 126.99a±15.17 | 5.078 | 0.0437 * |
| K00261 | *gdhA* | 180.08a±7.86 | 159.08a±2.33 | 149.35a±2.03 | 192.96a±2.15 | 206.96a±8.29 | 5.698 | 0.0343 * |
| K00117 | *gdhB* | 435.52b±28.77 | 668.94ab±8.18 | 557.04ab±13.76 | 811.23a±6.79 | 730.21a±24.83 | 17.9 | 0.00117 ** |
| K00284 | *gltB* | 145.58b±5.5 | 225.11ab±5.25 | 209.77ab±4.82 | 314.38a±25.18 | 248.6ab±12.51 | 11.97 | 0.00472 ** |
| K00266 | *gltD* | 203.56a±2.11 | 219.67a±9.96 | 205.19a±3.09 | 281.08a±25.1 | 240.83a±18.47 | 4.172 | 0.0637 |
| K01915 | *glnA* | 502.4a±10.39 | 474.41a±19.77 | 446.39a±0.11 | 583.86a±33.77 | 551.1a±57.93 | 2.367 | 0.15 |

**Table S10. Differences in abundance (mean ± SE) of** **selected microbial Phosphorus (P) cycling genes for all successional stage metagenomes.** Different letters indicate significant differences (ANOVA, *P* < 0.05, LSD test) among different successional stages. *, *P* < 0.05; **, *P* < 0.01; ***, *P* < 0.001.

| KEGG number | Gene name | S1 | S2 | S3 | S4 | S5 | F | P |
| --- | --- | --- | --- | --- | --- | --- | --- | --- |
| K02038 | *pstA* | 63.71c±3.32 | 93.5b±2.16 | 87bc±3.43 | 123.45a±1.72 | 135.59a±10.28 | 28.72 | 3.88e-05 *** |
| K02036 | *pstB* | 211.62ab±5.53 | 203.11b±10.73 | 215.04ab±4.21 | 283.52a±18.42 | 255ab±22.21 | 5.238 | 0.0185 * |
| K02037 | *pstC* | 74.81d±3.81 | 99.66bc±2.69 | 91.9cd±3.4 | 117.53ab±4.89 | 123.96a±5.64 | 21.24 | 0.000132 *** |
| K02040 | *pstS* | 97.06c±3.21 | 151.61a±3.39 | 126.38b±0.83 | 144.1ab±4.07 | 152.34a±5.77 | 33.61 | 2.02e-05 *** |
| K02041 | *phnC* | 20.17b±1.17 | 23.61b±1.07 | 20.1b±1.41 | 30.45b±1.23 | 52.29±6.76a | 15.63 | 0.000439 *** |
| K02044 | *phnD* | 21.1c±1.1 | 33.57bc±0.34 | 36.76bc±6.42 | 50.99b±6.15 | 110.38a±7.28 | 51.62 | 3.3e-06 *** |
| K02042 | *phnE* | 14.2b±0.63 | 16.51b±1.09 | 16.96b±1.77 | 26.21b±1.63 | 51.9a±6.87 | 20.46 | 0.000153 *** |
| K05814 | *ugpA* | 29.93b±3.01 | 37.46±ab2.25 | 44.82ab±5.65 | 54.82ab±7.19 | 63.43a±6.7 | 6.619 | 0.00909 ** |
| K05813 | *ugpB* | 32.89b±4.96 | 43.74ab±5.89 | 53.55ab±7.32 | 62.65ab±7.47 | 73.3a±6.13 | 6.564 | 0.00933 ** |
| K05816 | *ugpC* | 28.01a±1.42 | 33.38a±3.2 | 34.34a±5.93 | 38.11a±5.25 | 40.8a±6.11 | 1.204 | 0.373 |
| K05815 | *ugpE* | 46.4a±7.62 | 39.66a±5.24 | 44.31a±3.88 | 47.41a±4.73 | 54.04a±5.81 | 0.827 | 0.54 |
| K01507 | *ppa* | 83.98a±6.16 | 70.97a±3.06 | 71.22a±1.91 | 89.77a±7.79 | 77.6a±4.47 | 2.156 | 0.156 |
| K00937 | *ppk*1 | 318.24c±1.28 | 381.49bc±15.58 | 375.42bc±9.73 | 477.65ab±32.96 | 538.02a±50.18 | 8.816 | 0.00353 ** |
| K01524 | *ppx* | 161.42c±4.51 | 196.65c±1.02 | 187.79c±0.12 | 246.15b±5.85 | 300.91a±15.49 | 45.26 | 5.79e-06 *** |
| K00117 | *gcd* | 435.52d±35.23 | 668.94bc±10.02 | 557.05cd±19.47 | 811.23a±8.31 | 730.21ab±30.41 | 38.61 | 1.13e-05 *** |
| K19670 | *phnA* | 7.87b±0.71 | 11.58ab±1.59 | 16.96ab±3.46 | 15.7ab±2.42 | 20.55a±1.28 | 7.302 | 0.00663 ** |
| K06167 | *phnP* | 32.55a±1.42 | 40.24a±1.92 | 32.94a±5.02 | 30.56a±1.5 | 30.99a±1.13 | 3.933 | 0.0409 * |
| K03430 | *phnW* | 6.09c±0.45 | 30.31a±2.46 | 25.69ab±1.31 | 20.68b±0.63 | 18.59b±0.74 | 46.84 | 5e-06 *** |
| K01126 | *ugpQ* | 67.59b±5.27 | 96.51ab±3.11 | 84.06ab±9.84 | 124.32a±4.8 | 122.01a±13.06 | 9.86 | 0.0024 ** |
| K01077 | *phoA* | 39.65c±4.63 | 74.47ab±8.52 | 44.87bc±9.11 | 74.03ab±2.2 | 103.88a±5.24 | 18.41 | 0.000233 *** |
| K01113 | *phoD* | 131.73b±5.4 | 166.8a±3.67 | 146.43ab±5.58 | 157.71ab±4.39 | 168.91a±7.78 | 7.882 | 0.00516 ** |
| K09474 | *phoN* | 1.1a±0.42 | 2.7a±0.77 | 2.84a±1.03 | 2.06a±0.45 | 4a±0.44 | 3.313 | 0.0626 |
| K07657 | *phoB* | 469.06c±18.86 | 574.16abc±13.69 | 502.03bc±28.31 | 637.75ab±35.25 | 684.01a±30.16 | 11.6 | 0.00134 ** |
| K07658 | *phoP* | 336.37b±11.11 | 415.38ab±4.83 | 351.93ab±15.89 | 419.8ab±14.73 | 440.29a±29.01 | 6.765 | 0.00849 ** |
| K07636 | *phoR* | 317.86b±6.91 | 367.7ab3±10.15 | 314.99b±28.65 | 388.64ab±10.55 | 454.85a±36.57 | 7.361 | 0.00646 ** |

**Supplementary Methods**

**Measurement of soil and litter physicochemical properties**

Soil pH was determined in the laboratory using a pH monitor (Thermo Orion-868, MA, USA) with a 1:5 ratio of fresh soil to distilled water. Soil water content (SWC) was measured gravimetrically after 48-h desiccation at 105 °C. Soil bulk density (BD) was measured using a 100 cm^3^ stainless-steel cutting ring in the center of each plot. The measurement of soil textures was carried out using a fully automatic laser granulometry (LA-960S, HORIBA, Japan), with the results expressed as percentage contents of clay, silt, and sand. The concentrations of total carbon (TC) in soil were determined through dry combustion (Vario EL cube, Hanau, Germany). The determination of soil organic carbon (SOC) in the extracts was conducted using an automated total organic carbon analyzer (TOC-VWP, Shimadzu, Japan). Soils were suspended in water (at a ratio of 1:5 soil to water) for 30 minutes and then filtered through 0.45-μm membranes to assess the soil dissolved organic carbon (DOC). Soil NH_4_^+^ and NO_3_^−^ nitrogen were extracted by shaking the sample vigorously with 50 mL of 1.0 mol l^-1^ KCl for 20 minutes and were then analyzed using a continuous flow analytical system (SEAL Analytical AA3, Nordstedt, Germany). Soil total nitrogen (TN) and total phosphorus (TP) were analyzed using a continuous flow analytical system (SEAL Analytical AA3, Nordstedt, Germany) following H_2_SO_4_ addition for TN and HClO_4_-H_2_SO_4_ digestion for TP. The concentrations of total potassium (TK) in soil were assessed through flame photometry (FP640, INASA, China). Litter total potassium (LTK) was determined by flame photometry (FP640, INASA, China) following H_2_SO_4_-H_2_O_2_ digestion, whereas litter total phosphorus (LTP) and litter total nitrogen (LTN) were analyzed using a continuous flow analytical system (SEAL Analytical AA3, Nordstedt, Germany). The measurement of litter total carbon (LTC) was conducted using a Flash 2000 NC Analyzer (Thermo Scientific, MA, USA). The hemicellulose and cellulose content of litter were determined using sequential extraction with neutral and acidic detergents, followed by strong acid hydrolysis. Standard testing methods were adopted to measure the above soil-litter properties, as previously described [1-3].

**Metagenomic sequencing and analysis**

The flowchart of metagenomic processing methods and the basic metagenomic sequencing information are shown in **Fig. S1** and **Table S2**, respectively. In detail, quality control of the raw metagenomic sequences was performed with Fastp (v0.20.0, https://github.com/OpenGene/fastp) to remove the adapters, low-quality reads with a length < 50 bp and reads with an average quality score < 20 and N bases. The obtained clean reads were assembled into contigs using Megahit (parameters: kmer_min = 47, kmer_max = 97, step = 10) (v1.1.2, http://www.l3-bioinfo.com/products/megahit.html) with the optimal k-mer parameter. Subsequently, contigs with sequences > 500 bp were used for the prediction of open reading frames (ORFs) using MetaGene (v3.26, http://metagene.cb.k.u-tokyo.ac.jp/) and then translated into amino acid sequences using the NCBI translation table (http://www.ncbi.nlm.nih.gov/Taxonomy/ taxonomyhome.html/index.cgi?chapter=tgencodes#SG1). All the predicted genes were clustered into a nonredundant gene catalog using CD-HIT (v4.6.1, http://www.bio informatics.org/cd-hit/) with 95% identity and 90% coverage. Clean reads from each sample were mapped to the nonredundant gene catalog that was generated in the previous step, using SOAPaligner (v2.2.1, http://soap.genomics.org.cn/soapaligner.html), and the number of reads corresponding to functional genes in each sample was counted. The non-redundant gene catalog was searched against Kyoto Encyclopedia of Genes and Genomes (KEGG v94.2, http://www.genome.jp/kegg/) and the Carbohydrate-Active Enzyme database (CAZy v2021, http://www.cazy.org/) with Diamond (v0.8.35, https://github.com/bbuchfink/diamond) and blastp algorithm (v2.2.28+, http://blast.ncbi.nlm.nih.gov/Blast.cgi) for functional annotation. The targeted C, N and P cycle-related genes were filtered out based on the main pathways of C, N and P cycling that simultaneously emerged across all the metagenomic samples. Information on the selected functional genes is provided in **Table S3**, **Table S4**, **Table S5**, and **Table S6**. Finally, the taxonomic annotation for the subset of selected genes was processed with Diamond software and blastp algorithm against the NR database (v201607-12) in NCBI (ftp://ftp.ncbi.nlm.nih.gov/blast/db/). To assess the abundance of these genes, high-quality sequences from each sample were mapped onto the predicted gene sequences using Salmon (https://salmon.readthedocs.io/) and the TPM (transcripts per kilobase per million mapped reads) was used to normalize the abundance values in metagenomes.

**Selections of functional gene families for the carbon, nitrogen and phosphorus cycling**

First, based on previous studies by [4], we selected functional gene families encoding the enzymatic activities involved in the degradation of the plant-and microbial compounds degradation according to CAZy (**Table S3**). Then, after conducting an extensive and comprehensive literature search [5-13], we have curated a set of 58 genes encoding enzymes (with KO IDs) potentially associated with processes such as lignin depolymerization, oxidative stress responses, the breakdown of lignin-derived aromatic compounds (e.g., funneling and fission pathways), and adaptation to inhibitors originating from lignocellulosic materials. Supplementary information **Table S4** provides details for each enzyme, including EC numbers, KO IDs, names, and functions. Thirdly, based on previous studies [14-15], we selected functional gene families associated with microbial N cycles using the KEGG database (**Table S5**). Specifically, the gene families involved in the N cycling pathway include nitrification, denitrification, dissimilatory nitrate reduction to ammonium nitrogen (DNRA), and assimilatory nitrate reduction to ammonium nitrogen (ANRA), N_2_ fixation, N transport, and organic N metabolism. Finally, the targeted P cycle-related genes (**Table S6**) were retrieved based on published studies [16-19].

**References cited in supplementary methods**

1. Bao S. Soil and agricultural chemistry analysis. Beijing: China agriculture press; 2000.

2. Ma B, Wang H, Dsouza M, Lou J, He Y, Dai Z, et al. Geographic patterns of co-occurrence network topological features for soil microbiota at continental scale in eastern China. ISME J. 2016;10:1891-901.

3. McTiernan KB, Coûteaux M-M, Berg B, Berg MP, de Anta RC, Gallardo A, et al. Changes in chemical composition of Pinus sylvestris needle litter during decomposition along a European coniferous forest climatic transect. Soil Biol Biochem. 2003;35:801-12.

4. Ren C, Zhang X, Zhang S, Wang J, Xu M, Guo Y, et al. Altered microbial CAZyme families indicated dead biomass decomposition following afforestation. Soil Biol Biochem. 2021;160:108362.

5. Brink DP, Ravi K, Lidén G, Gorwa-Grauslund MF. Mapping the diversity of microbial lignin catabolism: experiences from the eLignin database. Appl Microbiol Biot. 2019;103:3979-4002.

6. Ceballos SJ, Yu C, Claypool JT, Singer SW, Simmons BA, Thelen MP, et al. Development and characterization of a thermophilic, lignin degrading microbiota. Process Biochem. 2017;63:193-203.

7. De Gonzalo G, Colpa DI, Habib MH, Fraaije MW. Bacterial enzymes involved in lignin degradation. J Biotechnol. 2016;236:110-9.

8. Díaz-García L, Bugg TD, Jiménez DJ. Exploring the lignin catabolism potential of soil-derived lignocellulolytic microbial consortia by a gene-centric metagenomic approach. Microb Ecol. 2020;80:885-96.

9. Janusz G, Pawlik A, Sulej J, Świderska-Burek U, Jarosz-Wilkołazka A, Paszczyński A. Lignin degradation: microorganisms, enzymes involved, genomes analysis and evolution. FEMS Microbiol Rev. 2017;41:941-62.

10. Kamimura N, Takahashi K, Mori K, Araki T, Fujita M, Higuchi Y, et al. Bacterial catabolism of lignin‐derived aromatics: new findings in a recent decade: update on bacterial lignin catabolism. Env Microbiol Rep. 2017;9:679-705.

11. Lin L, Wang X, Cao L, Xu M. Lignin catabolic pathways reveal unique characteristics of dye‐decolorizing peroxidases in Pseudomonas putida. Environ Microbiol. 2019;21:1847-63.

12. Xu Z, Lei P, Zhai R, Wen Z, Jin M. Recent advances in lignin valorization with bacterial cultures: microorganisms, metabolic pathways, and bio-products. Biotechnol Biofuels. 2019;12:1-19.

13. Zhu D, Si H, Zhang P, Geng A, Zhang W, Yang B, et al. Genomics and biochemistry investigation on the metabolic pathway of milled wood and alkali lignin-derived aromatic metabolites of Comamonas serinivorans SP-35. Biotechnol Biofuels. 2018;11:1-15.

14. Kelly CN, Schwaner GW, Cumming JR, Driscoll TP. Metagenomic reconstruction of nitrogen and carbon cycling pathways in forest soil: Influence of different hardwood tree species. Soil Biol Biochem. 2021;156:108226.

15. Tu Q, He Z, Wu L, Xue K, Xie G, Chain P, et al. Metagenomic reconstruction of nitrogen cycling pathways in a CO2-enriched grassland ecosystem. Soil Biol Biochem. 2017;106:99-108.

16. Dai Z, Liu G, Chen H, Chen C, Wang J, Ai S, et al. Long-term nutrient inputs shift soil microbial functional profiles of phosphorus cycling in diverse agroecosystems. ISME J. 2020;14:757-70.

17. Hu X, Gu H, Liu J, Wei D, Zhu P, Cui Xa, et al. Metagenomic strategies uncover the soil bioavailable phosphorus improved by organic fertilization in Mollisols. Agr Ecosyst Environ. 2023;349:108462.

18. Liang J-L, Liu J, Jia P, Yang T-t, Zeng Q-w, Zhang S-c, et al. Novel phosphate-solubilizing bacteria enhance soil phosphorus cycling following ecological restoration of land degraded by mining. ISME J. 2020;14:1600-13.

19. Wu X, Rensing C, Han D, Xiao K-Q, Dai Y, Tang Z, et al. Genome-resolved metagenomics reveals distinct phosphorus acquisition strategies between soil microbiomes. Msystems. 2022;7:e01107-21.
